# Co-Inflammatory Roles of TGFβ1 in the Presence of TNFα Drive a Pro-inflammatory Fate in Mesenchymal Stem Cells

**DOI:** 10.3389/fimmu.2017.00479

**Published:** 2017-05-11

**Authors:** Shalom Lerrer, Yulia Liubomirski, Alexander Bott, Khalid Abnaof, Nino Oren, Afsheen Yousaf, Cindy Körner, Tsipi Meshel, Stefan Wiemann, Adit Ben-Baruch

**Affiliations:** ^1^Faculty of Life Sciences, Department of Cell Research and Immunology, Tel Aviv University, Tel Aviv, Israel; ^2^Division of Molecular Genome Analysis, German Cancer Research Center (DKFZ), Heidelberg, Germany

**Keywords:** bone marrow-derived mesenchymal stem cells, NF-κB, pro-inflammatory mediators, Smad3, TGFβ1, TNFα

## Abstract

High plasticity is a hallmark of mesenchymal stem cells (MSCs), and as such, their differentiation and activities may be shaped by factors of their microenvironment. Bones, tumors, and cardiomyopathy are examples of niches and conditions that contain MSCs and are enriched with tumor necrosis factor α (TNFα) and transforming growth factor β1 (TGFβ1). These two cytokines are generally considered as having opposing roles in regulating immunity and inflammation (pro- and anti-inflammatory, respectively). Here, we performed global gene expression analysis of human bone marrow-derived MSCs and identified overlap in half of the transcriptional programs that were modified by TNFα and TGFβ1. The two cytokines elevated the mRNA expression of soluble factors, including mRNAs of pro-inflammatory mediators. Accordingly, the typical pro-inflammatory factor TNFα prominently induced the protein expression levels of the pro-inflammatory mediators CCL2, CXCL8 (IL-8), and cyclooxygenase-2 (Cox-2) in MSCs, through the NF-κB/p65 pathway. In parallel, TGFβ1 did not elevate CXCL8 protein levels and induced the protein expression of CCL2 at much lower levels than TNFα; yet, TGFβ1 readily induced Cox-2 and acted predominantly *via* the Smad3 pathway. Interestingly, combined stimulation of MSCs by TNFα + TGFβ1 led to a cooperative induction of all three inflammatory mediators, indicating that TGFβ1 functioned as a co-inflammatory cytokine in the presence of TNFα. The cooperative activities of TNFα + TGFβ1 that have led to CCL2 and CXCL8 induction were almost exclusively dependent on p65 activation and were not regulated by Smad3 or by the upstream regulator TGFβ-activated kinase 1 (TAK1). In contrast, the TNFα + TGFβ1-induced cooperative elevation in Cox-2 was mostly dependent on Smad3 (demonstrating cooperativity with activated NF-κB) and was partly regulated by TAK1. Studies with MSCs activated by TNFα + TGFβ1 revealed that they release factors that can affect other cells in their microenvironment and induce breast tumor cell elongation, migration, and scattering out of spheroid tumor masses. Thus, our findings demonstrate a TNFα + TGFβ1-driven pro-inflammatory fate in MSCs, identify specific molecular mechanisms involved, and propose that TNFα + TGFβ1-stimulated MSCs influence the tumor niche. These observations suggest key roles for the microenvironment in regulating MSC functions, which in turn may affect different health-related conditions.

## Introduction

Mesenchymal stem cells (MSCs) are characterized by high plasticity and have critical roles in regulating physiological and pathological processes, in health and disease (1–3). MSC differentiation into different lineages and their versatile activities reflect, among others, their response to microenvironment cues residing at specific niches. Among the signals regulating the migration patterns taken by MSCs and their functional diversification are cytokines, which typically are regarded as key regulators of acquired immunity or inflammation (4, 5). Bone marrow (BM)-derived MSCs share the bone niche with hematopoietic cells and with their products and often encounter immune/inflammatory modulators in remote organs following their migration to these sites (4, 5). The interactions of MSCs with immune and inflammatory cells and with the factors they release may have a strong impact on the way the MSCs then affect their surrounding microenvironment.

While the understanding of MSC regulation by their intimate microenvironments has been improved recently (5–8), much is yet to be revealed. Here, we aimed to unravel the regulation of MSC phenotypes and functions by cytokines that are typically present at MSC niches (4, 5). Particularly, we determined the fate of BM-derived MSCs upon exposure to tumor necrosis factor α (TNFα) and transforming growth factor β1 (TGFβ1) that have been associated with opposing roles in immune and inflammatory activities; these two cytokines are coexpressed in specific niches also harboring MSCs (4, 5, 9, 10).

TNFα is a strong pro-inflammatory cytokine that has key roles in promoting leukocyte recruitment to injured/infected sites through induction of expression of adhesion molecules and of inflammatory chemokines (9, 11–13). The nature of TGFβ1 is more complex: in the presence of IL-1β + IL-6/IL-21/IL-23, TGFβ1 can promote Th17-mediated pro-inflammatory responses; yet, TGFβ1 is mostly identified as a very potent anti-inflammatory and immunosuppressive cytokine, opposing the activities of TNFα, inducing the generation of T regulatory cells, and mediating the anti-inflammatory activities exerted by such cells (10–15).

Despite their general opposing roles in immune regulation, TNFα and TGFβ1 coexist and act simultaneously in specific niches, where their joint activities may also influence the fate of MSCs and their respective functions. One such example is the bone, where macrophage-derived TNFα and TGFβ1 induce the migration of MSCs and their differentiation to osteoblasts, thus promoting bone formation (16, 17). Recent findings suggest that reduced bone-forming activities in MSCs are connected to excessive inflammatory conditions that are ensued with increased age, possibly reflecting changes in the microenvironment and its cytokine contents, which may include TNFα and TGFβ1 (6, 18, 19).

The tumor microenvironment (TME) provides another example for potential coregulation of MSC activities by TNFα and TGFβ1 (20–23). While in pathogen-induced immunity, TGFβ1-mediated suppression may follow TNFα-driven inflammatory processes and shut them off, in malignancy the two processes coexist and eventually they both promote disease progression (20–26). Recent published reports indicate that the pro-tumoral activities of TNFα and TGFβ1 are manifested through their impact on the cancer cells and on cells of the TME, such as MSCs that populate the tumors (27–32). In response to TNFα, MSCs gain a pro-inflammatory phenotype that drives forward the metastatic cascade (20, 21, 28, 33, 34). In parallel, TGFβ1-stimulated MSCs release factors that act directly on tumor cells and promote their invasive properties (31, 32). Moreover, a recent report demonstrated that the tumor-enhancing activities of TNFα-primed adipose tissue-derived MSCs are mediated by TGFβ1, suggesting close interactive relationships between these two seemingly opposing cytokines (27).

These studies have led us to hypothesize that in microenvironments containing both TNFα and TGFβ1, the two cytokines regulate MSC functions through separate/shared mechanisms and that as a result of such molecular effects, the MSCs then affect cells at their intimate surroundings. The results of our current study indeed support this hypothesis. We demonstrate that in the presence of TNFα, TGFβ1 expressed pro-inflammatory activities and that jointly the two cytokines have increased the pro-inflammatory phenotype of BM-derived MSCs more than each cytokine alone. This was evidenced by increased protein levels of CCL2, CXCL8 (IL-8), and cyclooxygenase-2 (Cox-2), which are well identified as strong pro-inflammatory factors (35–39). This cooperativity between TNFα + TGFβ1 reflected channeling of their signals to different molecular paths: activation of NF-κB regulated the induction of CCL2 and CXCL8 while activation of Smad3 played a major role in inducing Cox-2 elevation. Our findings also identified divergent roles for the pathway of TGFβ-activated kinase 1 (TAK1) in regulating TNFα + TGFβ1-induced CCL2/CXCL8, compared to Cox-2-induced expression, in the MSCs. Of note, as a consequence of the joint activities of TNFα + TGFβ1 stimulation, the MSCs released factors that have led to elevated migratory and scattering processes in breast tumor cells.

Taken together, the findings of the current study demonstrate the functional relevance of the microenvironment in shaping the functions of MSCs and provide a proof of concept to the notion that TNFα + TGFβ1-stimulated MSCs affect their surroundings. These findings can contribute to an improved understanding of the way MSCs are regulated by the microenvironment and the way they impact their intimate milieu, demonstrating potential relevance of such events to physiological and pathological conditions in which MSCs are key tissue determinants.

## Materials and Methods

### Origin and Growth of MSCs

Human BM-derived MSCs were purchased from Lonza (Cat# PT-2501; Lonza, Walkersville, MD, USA). The cells were validated as MSCs by Lonza, by marker criteria (positive for CD44, CD29, CD105, and CD166; negative for CD45, CD14, and CD34) and differentiation to adipogenic, chondrogenic, and osteogenic lineages. MSCs of six different donors were used in the study. The cells were thawed in MSC growth medium (MSCGM; Cat# PT-3001; Lonza) and then were subcultured every 5–7 days, for up to 10 passages, in MSCGM or enriched Dulbecco’s modified Eagle’s medium (DMEM; Biological Industries, Beit Ha’emek, Israel), including 10% fetal bovine serum (FBS), 100 U/ml penicillin, 100 µg/ml streptomycin, 250 ng/ml amphotericin, and 4 mM l-glutamine (all from Biological Industries).

### MSC Stimulation

Following overnight incubation in “experimental medium” (DMEM containing the above-mentioned supplements without FBS, or with 0.5% FBS, as appropriate for experimental conditions; see figure legends), MSCs were stimulated with TNFα (50 ng/ml; Cat# 300-01; PeproTech, Rocky Hill, NJ, USA) and/or TGFβ1 (10 ng/ml; Cat# 100-21; PeproTech). TNFα concentration was selected based on our previous studies (40–43), as well as other cell systems (e.g., Ref. 44–46). TGFβ1 concentration was selected based on a literature search (47–49) and preliminary titration analysis (data not shown). In all procedures, control non-stimulated cells were treated with the diluents of the cytokines (= vehicle control). In array experiments, MSCs were stimulated with TNFα or TGFβ1 for 1, 3, 7, 14, and 24 h. In signaling experiments, the stimulation time was 10 min, and in functional assays it was 24 h.

### Illumina Beadchip Array Analyses

#### Processing and Normalization

Following MSC stimulation by the cytokines (as described above), total-RNA of frozen cell pellets was isolated using miRNeasy Mini kit (Cat# 217004; Qiagen, Hilden, Germany) according to manufacturer’s protocols. The quality of total RNA was checked by gel analysis using the total RNA Nano chip assay on an Agilent 2100 Bioanalyzer (Agilent Technologies GmbH, Berlin, Germany). RNA concentrations were determined using the NanoDrop spectrophotometer (NanoDrop Technologies, Wilmington, DE, USA).

#### Genome-Wide Gene Expression Profiling

This step was performed using HumanHT-12 v4 BeadChips (Illumina, San Diego, CA, USA) in the Genomics and Proteomics Core Facility at the German Cancer Research Center (DKFZ), Heidelberg, Germany. Hybridization was performed at 58°C, in GEX-HCB buffer (Illumina Inc.) at a concentration of 100 ng cRNA/μl, unsealed in a wet chamber for 20 h. Spike-in controls for low-, medium-, and highly abundant RNAs were added, as well as mismatch control and biotinylation control oligonucleotides. Raw probe intensities were extracted, background-corrected, normalized, and summarized to expression levels using the variance stabilization normalization method (50). The complete dataset was deposited at ArrayExpress (51, 52) (accession numbers E-MTAB-5421 and E-MTAB-5420).

#### Gene Ontology (GO) Analysis

**Figure 1:**
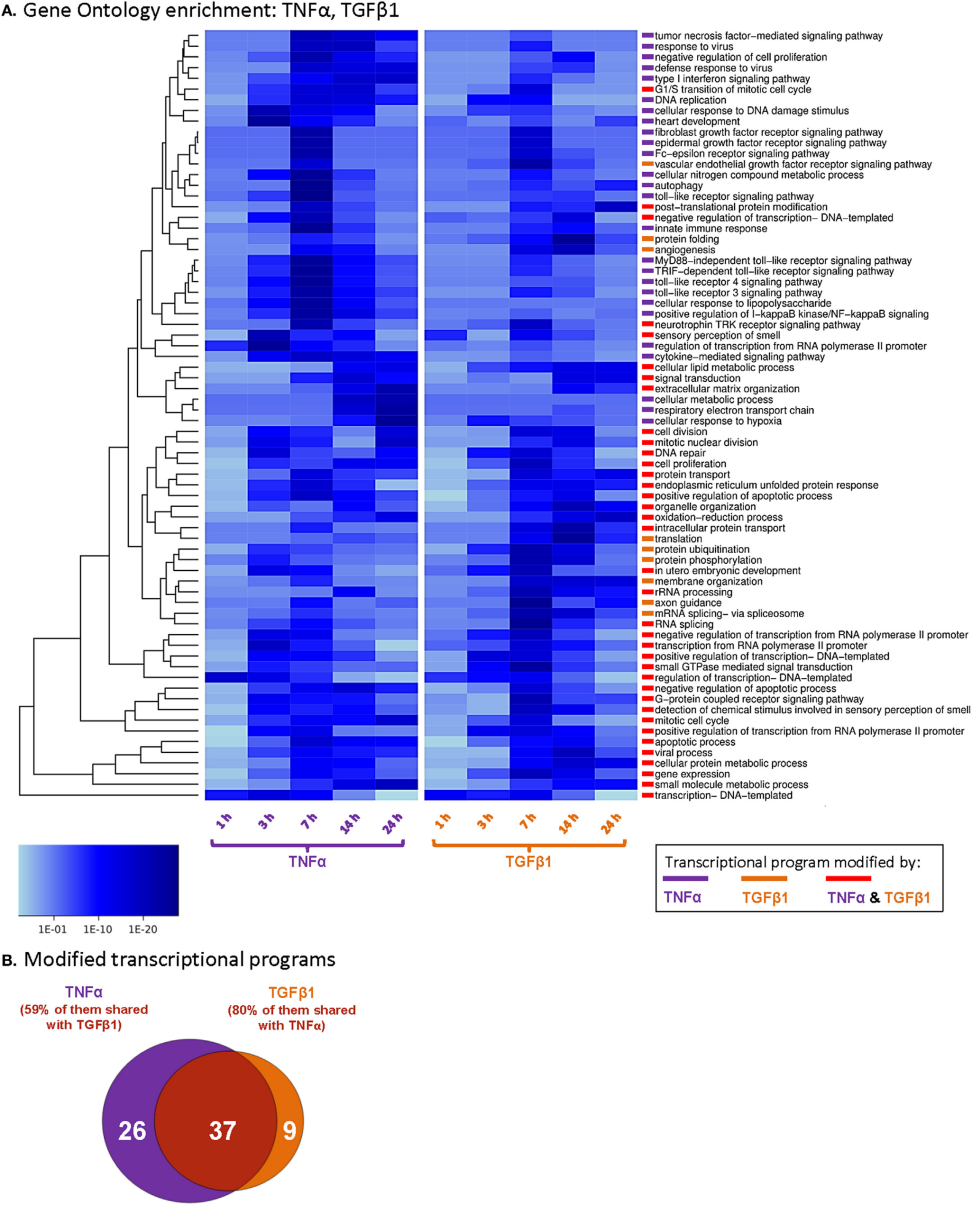
**TNFα and TGFβ1 modify private and shared transcriptional programs in MSCs**. Human BM-derived MSCs of Donor #1 were stimulated by TNFα (50 ng/ml) or TGFβ1 (10 ng/ml) or treated by a vehicle control, as illustrated in the experimental design of Figure S1A in Supplementary Material (cytokine concentrations were selected as described in Section “Materials and Methods”; experiment performed in FBS-free medium). RNA was subjected to Illumina Beadchip array analyses, and the complete dataset was deposited at ArrayExpress [(51, 52); accession numbers E-MTAB-5421 and E-MTAB-5420]. **(A)** The figure presents the transcriptional programs modified in MSCs at different time points following their stimulation by TNFα or TGFβ1. Data are presented by *p*-value scaling (*p* ≤ 0.001 after Benjamini–Yekutieli correction for multiple testing). All the transcriptional programs that are demonstrated include ≥10 genes. **(B)** Venn diagram showing the number of transcriptional programs significantly affected only by TNFα, only by TGFβ1, or by both cytokines.

All differentially expressed genes were scanned at each time point for enrichment in the gene sets of the GO project terms (53). Enrichment analysis was performed for the different time points individually by taking the negative value of the logarithm of uncorrected *p*-values [−log (*p*-value)] as ranking scores for the transcript. Gene sets of GO terms were then tested for their association with these ranking scores *via* a univariate logistic regression-based method as described in the studies of Sartor et al. (54) and Montaner and Dopazo (55). Resulting *p*-values of GO terms were then adjusted according to Benjamini–Yekutieli’s method for false discovery rate (FDR) control under dependency (56). Significant GO terms are reported at a cutoff value of FDR ≤ 0.001 in Figure 1 and at a cutoff value of FDR ≤ 0.01 in Table S1 in Supplementary Material.

#### Differential Gene Expression Analyses

These analyses were performed *via* the “Limma” method (57, 58) that uses linear models and empirical Bayes. At 1, 3, 7, 14, and 24 h after stimulation (TNFα or TGFβ1), sample sets of each stimulation were compared to their counterpart vehicle-treated control cells (0 and 24 h). Statistical dependencies of samples within time points and replicates were considered *via* a factorial design matrix in “Limma”. Corrections for multiple testing were performed using Benjamini–Hochberg’s method (59), and significant differentially expressed genes were reported at a cutoff value of FDR ≤ 0.005 and absolute log_2_ fold change ≥ 1.5 (= fold change ≥ 2.8).

### Quantitative Real-time Polymerase Chain Reaction (qPCR)

Following global profiling, the upregulated expression of mRNAs was validated by qPCR analysis, at the 3–14-h range, following MSC stimulation. Two procedures were used: (1) quantification of PTGS2, CX3CL1, EPSTI1, ANGPTL4, PTHLH, and PLAU expression levels: total RNA was isolated using the EZ-RNA kit (Cat# 20-400; Biological Industries). RNA samples were used for generation of first-strand complementary DNA synthesis using the M-MLV reverse transcriptase (Cat# AM2044; Ambion, Austin, TX, USA). Quantification of cDNA targets by qPCR was performed on Rotor Gene 6000 (Corbett Life Science, Concorde, NSW, Australia). Transcripts were detected using Absolute Blue qPCR SYBR Green ROX mix (Cat# AB-4163/A; Thermo Fisher Scientific, Waltham, MA, USA) according to manufacturer’s instructions. The sequences of the primers are listed in Table S2A in Supplementary Material. In each reaction, two pairs of specific primers were used, which had been designed to span different exons. Data were normalized to the housekeeping gene RPS9. Dissociation curves for each primer set indicated a single product after the 40 cycles used for analysis (except for CX3CL1: 50 cycles), and no-template controls were negative. Quantification was performed by standard curves, within the linear range of quantification. (2) Quantification of CCL2, CXCL8, NGF, IL6, LIF, HBEGF, CSF2, MMP1, MMP3, VEGFC, FGF1, and IL12A expression levels: mRNAs were isolated using miRNeasy Mini kit (Qiagen, Hilden, Germany) according to manufacturer’s instructions. cDNA synthesis was performed with Revert Aid H Minus first Strand cDNA Synthesis Kit (Thermo Fisher Scientific), and qPCR amplifications of specific genes were performed in an ABI Prism 7900HT Sequence Detection System (Applied Biosystems, Foster City, CA, USA). Probes from Universal Probe Library (UPL; Roche Diagnostics GmbH, Mannheim, Germany) were used to increase primer specificity. Analysis was performed by using 2^−ΔΔCT^. The sequences of the primers and the UPL probes used are listed in Table S2B in Supplementary Material. Data were normalized to the housekeeping genes GAPDH and HPRT.

### Western Blotting

Following MSC stimulation by the cytokines (as described above), the cells were lysed in RIPA lysis buffer and conventional Western blot (WB) procedures were performed, using antibodies (Abs) directed against the following proteins: phosphorylated (P)-p65 [Cat# 3033; Cell Signaling Technology (CST), Danvers, MA, USA]; total (T)-p65 (Cat# 4764 or Cat# 8242; CST); IκBα (Cat# 4814; CST); P-Smad3 (Cat# 9520; CST); T-Smad3 (Cat# 9523; CST); T-TAK1 (Cat# 4505; CST); Cox-2 (Cat# PA1725; Boster Immunoleader, Pleasanton, CA, USA); Abs directed against GAPDH (Cat# ab9485; Abcam, Cambridge, UK); or β-tubulin (Cat# ab6046; Abcam) served for loading controls. Then, membranes were reacted with horseradish peroxidase (HRP)-conjugated goat anti-rabbit IgG or HRP-conjugated goat anti-mouse IgG, as appropriate (Cat# 111-035-003; Cat# 115-035-071, respectively; Jackson ImmunoResearch Laboratories, West Grove, PA, USA), subjected to enhanced chemiluminescence (Cat# 20-500; Biological Industries), and visualized using Kodak Medical X-RAY processor (Carestream Health, Rochester, NY, USA).

### ELISA Assays

Following MSC stimulation by the cytokines (as described above), cell conditioned media (CM) were collected and cleared by centrifugation. Extracellular expression levels of CCL2 and CXCL8 in CM were determined by ELISA, using standard curves at the linear range of absorbance with recombinant human (rh) CCL2 and CXCL8 (Cat# 300-04 and #200-08M, respectively; PeproTech). The following Abs were used (all from PeproTech): for CCL2: coating mouse monoclonal Abs (Cat# 500-M71); detecting biotinylated rabbit polyclonal Abs (Cat# 500-P34Bt). For CXCL8: coating rabbit polyclonal Abs (Cat# 500-P28); detecting biotinylated rabbit polyclonal Abs (Cat# 500-P28Bt). After the addition of HRP (Cat# 016-030-084; Jackson ImmunoResearch Laboratories), the substrate TMB/E solution (Cat# ES001; Millipore, Temecula, CA, USA) was added, the reaction was stopped by addition of 0.18 M H_2_SO_4_, and absorbance was measured at 450 nm.

### Analysis of METABRIC and TCGA Patient Datasets

The correlation between expression levels of CCL2, CXCL8 and PTGS2 (Cox-2) in patients expressing high/low levels of TNFα + TGFβ1 was performed using gene expression data from the RNA-Seq-based TCGA dataset, including data from 1,215 breast cancer patients (60). Here, patients were divided into quartiles based on the expression levels of TNFα and of TGFβ1. Patients were individually assigned to low expression (i.e., lower quartile) or high expression (upper quartile) of each cytokine. Expression of the target genes in patients exhibiting high expression of both cytokines, low expression of both cytokines, or high expression of one and low expression of the other was illustrated in box plots. Patients belonging to the second and third quartiles for one of the cytokines were not considered in this analysis. Patient numbers in the different groups were as follows: TNFα-high + TGFβ1-high = 85 patients; TNFα-high + TGFβ1-low = 33 patients; TNFα-low + TGFβ1-high = 48 patients; and TNFα-low + TGFβ1-low = 105 patients. Statistical analysis was performed with two-tailed Mann–Whitney test.

The TCGA dataset was used also to determine the associations between the expression levels of CCL2, CXCL8 and PTGS2 (Cox-2) in the patient cohort. A similar analysis was performed with the METABRIC dataset (61), including data from 1,992 breast cancer patients. The following probes were used: CCL2 ILMN_1720048; CXCL8 ILMN_2184373; and PTGS2 (Cox-2) ILMN_2054297. In both datasets, log_2_-transformed expression values were outlined as scatter plots. Correlation coefficients and *p*-values were analyzed using Spearman correlation.

### Transfection of siRNAs in MSCs

Transient siRNA transfections were performed using the Lipofectamine RNAiMAX transfection reagent (Cat# 56531; Invitrogen, Grand Island, NY, USA) according to the manufacturer’s instructions, with the following siRNAs (all from Dharmacon, Lafayette, CO, USA): p65 siRNA pool (Cat# L-003533-00); Smad3 siRNA pool (Cat# L-020067-00); TAK1 siRNA pool (Cat# L-003790-00); and non-targeting control siRNA pool (Cat# D-001810-10). After 16 h, the medium was replaced with experimental medium (described above) for additional 24–48 h, and the cells were then stimulated by the cytokines as described above.

### Breast Tumor Cell Cultures

The human breast tumor cell lines MDA-MB-231 (HTB-26™) and MCF-7 (HTB-22™) were obtained from ATCC (Manassas, VA, USA) and grown in enriched DMEM. To generate mCherry-expressing MDA-MB-231 and MCF-7 cells, two rounds of retroviral infections were performed as previously described (42), with minor technical adaptations. Seventy-two hours following the second infection, infected cells were selected with 1 µg/ml (MDA-MB-231) or 4 µg/ml (MCF-7) of puromycin (Cat# P-1033; A.G. Scientific, San Diego, CA, USA) for 7 days.

### Stimulation of Breast Tumor Cells with MSC-Derived CM: Morphology and Migration Assays

mCherry-expressing breast tumor cells were plated in enriched DMEM medium for 24 h, then the medium was replaced by the following: (1) control medium; (2) medium containing TNFα + TGFβ1 at the same concentrations used for MSC stimulation (as above); (3) CM derived from vehicle-stimulated MSCs; and (4) CM derived from TNFα + TGFβ1-stimulated MSCs. The media of Groups 1 and 2 were kept in the same conditions as MSC-derived CM of Groups 3 and 4. All media were filtered through a 0.45 µm membrane prior to addition to the tumor cells. Following addition of media from Groups 1–4 to MDA-MB-231 cells (for 48–72 h) and to MCF-7 cells (for 48 h), morphology was determined by fluorescent microscopy. Transwell migration of MCF-7 cells was performed in inserts with 8 µm pore size (Cat# 3422; Corning, Cambridge, MA, USA), in which the upper compartment of the inserts was precoated with fibronectin (20 µg/ml, diluted in serum-free DMEM; Cat# 03-090-1; Biological Industries) for 1 h at 37°C. The inserts were placed in new wells, containing DMEM supplemented with 10% FBS in the lower compartment. A total of 1 × 10^5^ viable MCF-7 cells (pretreated for 48 h with the different MSC-derived CM or the respective control media, as described above) were added to the upper compartment of the inserts in serum-free DMEM. Following 21–22 h of incubation, the cells on the upper surface of the insert were removed, and the filters were fixed in ice-cold methanol and stained with Hemacolor (Cat# 1.11661; Merck, Darmstadt, Germany). Migrating cells were photographed at ×40 magnification and counted. Data are presented as number of cells in five fields that cover most of the insert.

### Stimulation of Breast Tumor Cells with MSC-Derived CM: Tumor Spheroid Assays

Six-well plates were incubated overnight on a rocker with 1.2% poly(2-hydroxyethyl methacrylate) (Cat# P3932; Sigma) diluted in ethanol. mCherry-expressing MCF-7 cells were plated in the coated wells, in DMEM/F12 medium supplemented with 2 mM l-glutamine, 100 U/ml penicillin, 100 µg/ml streptomycin, 250 ng/ml amphotericin (all from Biological Industries), 0.4% BSA (Cat# 0332-TAM; Amresco, Solon, OH, USA), B-27 serum-free supplement (Cat# 17504044; Gibco, Life technologies, Grand island, NY, USA), 20 ng/ml rh-basic FGF (Cat# 100-18B; PeproTech), 20 ng/ml rh-EGF (Cat# 236-EG; R&D systems, Minneapolis, MN, USA), and 5 µg/ml insulin (Cat# I9278; Sigma). After 72 h, tumor spheroids were collected, centrifuged (1,200 rpm for 7 min, + 4°C) and resuspended in the different MSC-derived CM or the respective control media (as described above). Tumor spheroids were photographed daily using fluorescent microscopy.

### Data Presentation and Statistical Analyses

The statistical analyses of mRNA arrays and METABRIC/TCGA analyses were described in their respective sections. Other *in vitro* experiments were performed in *n* ≥ 3 independent experimental repeats, with MSCs from ≥ 2 different donors, as indicated in respective figure legends. Data of TNFα + TGFβ1-induced functional assays with siRNAs are presented in two biological replicates, one in the main body of the manuscript and one in Supplementary Material. The results of ELISA and migration assays were compared by two-tailed unpaired Student’s *t*-test. Values of *p* ≤ 0.05 were considered statistically significant.

## Results

### TNFα and TGFβ1 Induce Different, yet Partly Overlapping Transcriptome Signatures in MSCs

To determine the impact of TNFα and TGFβ1 on BM-derived MSCs, we performed genome-wide expression analysis, in which we identified mRNAs that were upregulated or downregulated in response to each of the two cytokines. To this end, MSCs of Donor #1 were exposed to TNFα (50 ng/ml) or TGFβ1 (10 ng/ml) for 1, 3, 7, 14, and 24 h or to vehicle control (0 and 24 h), in two biological replicates (Figure S1A in Supplementary Material). Following Illumina Beadchip analysis, unsupervised clustering of total-mRNA expression proved high reproducibility between replicates (Figure S1B in Supplementary Material). The analyses demonstrated that: (1) all four vehicle samples (two of time “0 h” and two of time “24 h”) were clustered together indicating that no changes had occurred in unperturbed conditions; (2) the 1-h samples of each of the cytokines clustered outside of the vehicle samples, indicating that differential regulation of genes is evident already at this early time point; and (3) each cytokine induced, in a kinetics-dependent manner, a private transcriptional program.

Then, GO enrichment analysis was performed, identifying transcriptional programs that were modified at the different time points by TNFα or TGFβ1. In this analysis, we focused on biological processes that include 10 or more genes (*n* ≥ 10), with significance of *p* ≤ 0.001 (compared to vehicle-stimulated cells; Figure 1A). TNFα stimulation modified the expression of 63 programs and TGFβ1 of 46 programs (Figures 1A,B). Altogether, we identified 72 programs that were modified by the two cytokines, with 37 programs overlapping between TNFα and TGFβ1 (51%; Figures 1A,B). Some of the processes that were modified by both cytokines reflected the potential impact of TNFα- and TGFβ1-stimulated MSCs on their microenvironment, including pathways such as oxidation–reduction processes, angiogenesis, and extracellular matrix organization (Table S1 in Supplementary Material).

**Figure 2:**
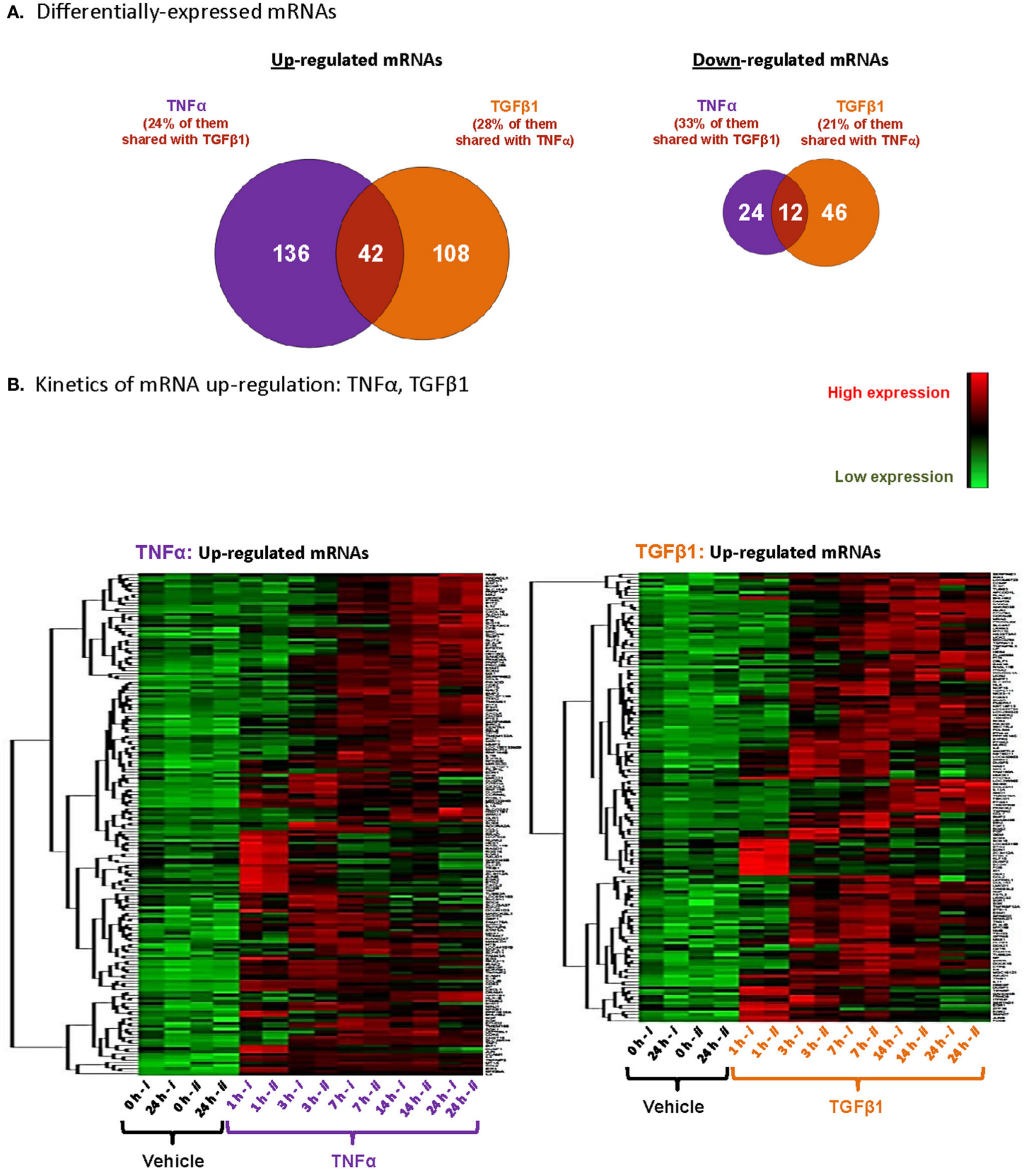
**TNFα and TGFβ1 induce private and shared modifications of mRNA expression in MSCs, in a time-dependent manner**. Based on the array analyses described in Figure 1 with MSCs of Donor #1, total numbers of upregulated and downregulated mRNAs were determined. **(A)** Venn diagram, demonstrating the patterns of gene regulation in MSCs stimulated by TNFα or TGFβ1. The figure includes mRNAs modified by log_2_ fold change ≥ 1.5 (= fold change ≥ 2.8) with *p* ≤ 0.005 after Benjamini–Hochberg correction for multiple testing, compared to vehicle-treated cells. A specific gene was considered as up- or downregulated if it has passed these cutoffs at one of the time points included in the analyses. **(B)** Visualization of upregulated mRNAs after unsupervised clustering, using the same criteria as in panel **(A)**. Each column represents a single replicate of each specific treatment (i, ii), and each row demonstrates a single upregulated mRNA.

Analysis of TNFα- or TGFβ1-deregulated mRNAs (cutoff: log_2_ fold change ≥ 1.5 = fold change ≥ 2.8; *p* ≤ 0.005) had identified a total of 178 mRNAs that were upregulated and 36 mRNAs that were downregulated following TNFα stimulation (in at least one of the time points; Figure 2A). After TGFβ1 stimulation, 150 mRNAs were upregulated and 58 mRNAs were downregulated (Figure 2A). Interestingly, a substantial proportion of the upregulated mRNAs—24% of those affected by TNFα and 28% of those affected by TGFβ1—were shared between the two cytokines. Shared downregulated mRNAs were also identified: 33% of TNFα-regulated genes and 21% of TGFβ1-regulated genes.

**Figure 3:**
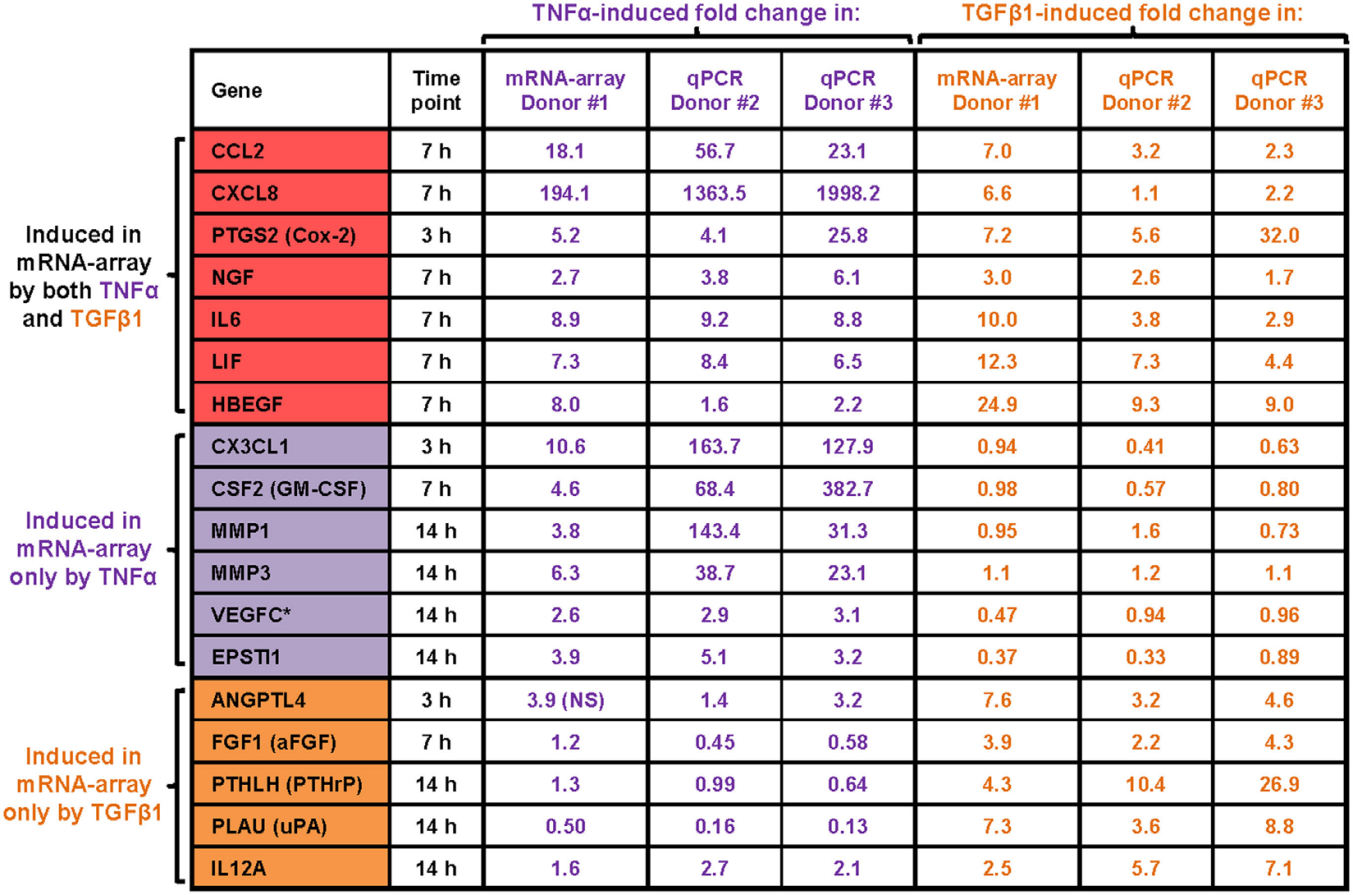
**qPCR validations of TNFα- or TGFβ1-upregulated mRNAs**. Following MSC stimulation by TNFα or TGFβ1 and the global gene analysis described in previous figures, upregulation of 18 selected mRNAs was validated. All 18 mRNAs were modified in the original array analysis by log_2_ fold change ≥ 1.5 (= fold change ≥ 2.8; * = fold change 2.6) with *p* ≤ 0.005 (after Benjamini–Hochberg correction for multiple testing, compared to vehicle-treated cells). The qPCR validations were performed with MSCs of two different donors (Donor #2 and Donor #3). The 18 selected mRNAs were validated by qPCR at time points in which they have undergone significant changes in the array analysis.

Furthermore, kinetics analysis demonstrated that major time-dependent alterations in transcriptional programs were induced after 3–14 h of stimulation by TNFα or TGFβ1 (Figure 2B). We selected 18 upregulated mRNAs to follow up on (Figure 3), focusing on secreted factors and pro-inflammatory mediators that can potentially impact the microenvironment of MSCs. Of these 18 mRNAs, six were induced only by TNFα, five were induced only by TGFβ1, and seven were induced by both cytokines. Increased mRNA expression of these 18 genes was validated by qPCR in MSCs from two additional donors—Donors #2 and #3 (Figure 3)—thus confirming the original array findings obtained in MSCs of Donor #1 (described above).

**Figure 4:**
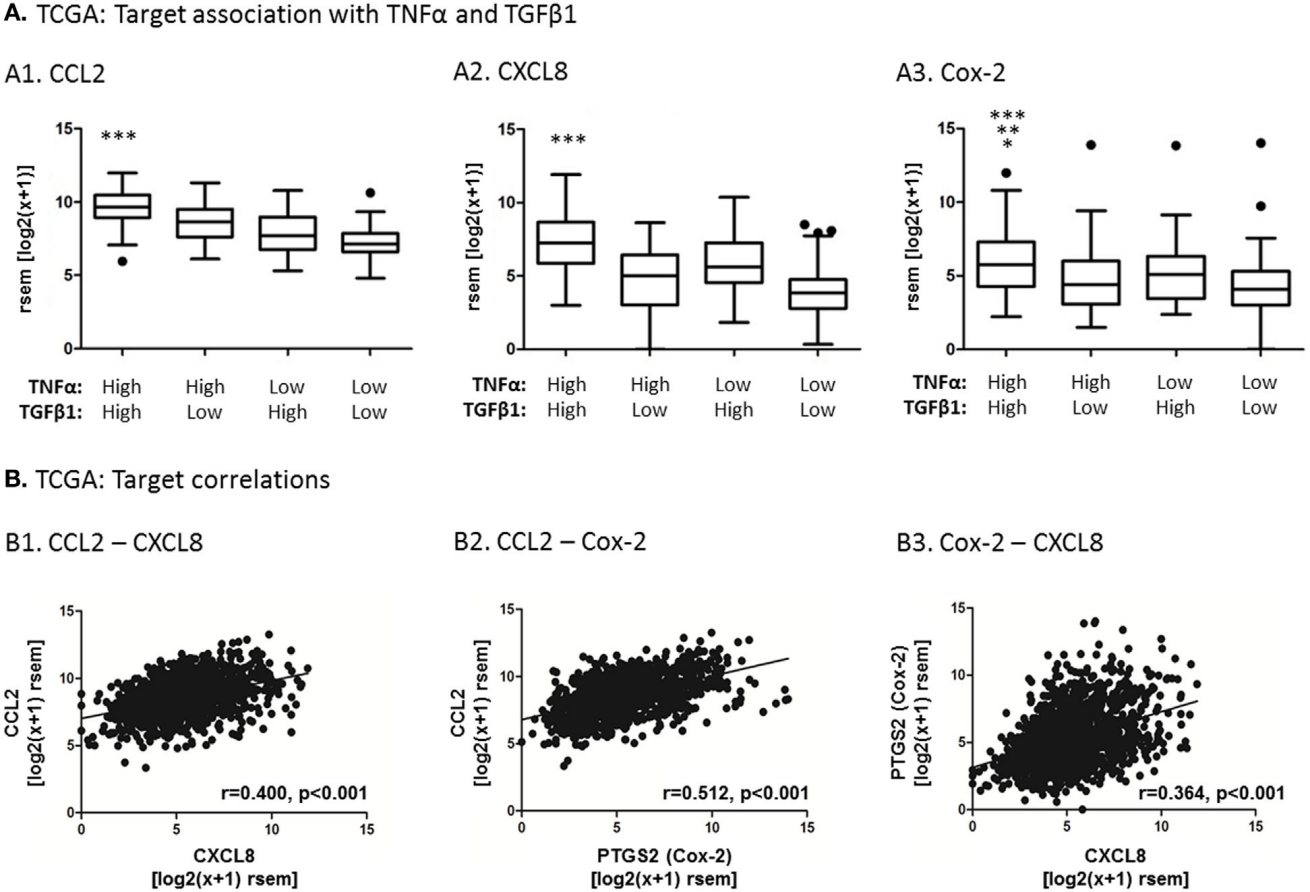
**Analyses of human breast tumors demonstrate coordinated expression of CCL2, CXCL8 and Cox-2, and higher levels of their expression in tumors enriched with both TNFα and TGFβ1**. Patient data analysis was performed with the TCGA dataset of human breast cancer, including 1,215 patient samples. **(A)** Associations between CCL2 (A1), CXCL8 (A2), Cox-2 (A3) expression and expression levels of TNFα and TGFβ1 in the tumors, performed by quartile analysis, as described in Section “Materials and Methods”. The black dots above the plots are outliers that are outside of the whiskers. Statistical analysis was performed by two-tailed Mann–Whitney test. In panel A1, ***p < 0.001 for CCL2 expression levels in the TNFα-high + TGFβ1-high patient group, compared to all other patient groups. In panel A2, ***p < 0.001 for CXCL8 expression levels in the TNFα-high + TGFβ1-high patient group, compared to all other patient groups. In panel A3, ***p < 0.001 for Cox-2 expression levels in the TNFα-high + TGFβ1-high patient group, compared to the TNFα-low + TGFβ1-low group, **p < 0.01 compared to the TNFα-high + TGFβ1-low group and *p < 0.05 compared to the TNFα-low + TGFβ1-high group. **(B)** Associations between CCL2, CXCL8 and Cox-2 expression levels in the patient cohort. The correlation coefficient (*r*) and statistical significance between two mRNAs in each graph were determined by Spearman correlation analysis. rsem, RNA-Seq by Expectation Maximization. Similar analysis performed with the METABRIC dataset of patient samples is demonstrated in Figure S2 in Supplementary Material.

To add clinical relevance to target selection, we analyzed the TCGA and METABRIC datasets of breast cancer patients, which included data from 1,215 patients and 1,992 patients, respectively. First, the TCGA dataset demonstrated that the expression of three pro-inflammatory genes, which are well established as tumor-promoting factors in breast cancer (37–39, 62–64)—CCL2, CXCL8 and PTGS2 that codes for Cox-2—was significantly elevated in breast tumors that expressed high levels of both TNFα and TGFβ1 together [Figure 4A; similar analyses could not be performed with the METABRIC dataset because TNFα and TGFβ1 (mostly the latter) were not properly detected in the original array that generated the dataset]. Moreover, in both the TCGA and the METABRIC datasets, the expression levels of CCL2, CXCL8 and PTGS2 were highly coregulated with each other in patient breast tumors (TCGA: Figure 4B; METABRIC: Figure S2 in Supplementary Material). Together, these findings suggest that tumors containing high levels of TNFα and TGFβ1 are enriched with the inflammatory and tumor-promoting mediators CCL2, CXCL8 and Cox-2, and that all three inflammatory mediators are coregulated in human breast tumors. Thus, our findings propose that since TNFα and TGFβ1 often coreside at the breast TME (20–23), their joint expression in tumors may induce the expression of CCL2, CXCL8 and Cox-2 in intratumoral MSCs.

**Figure 5:**
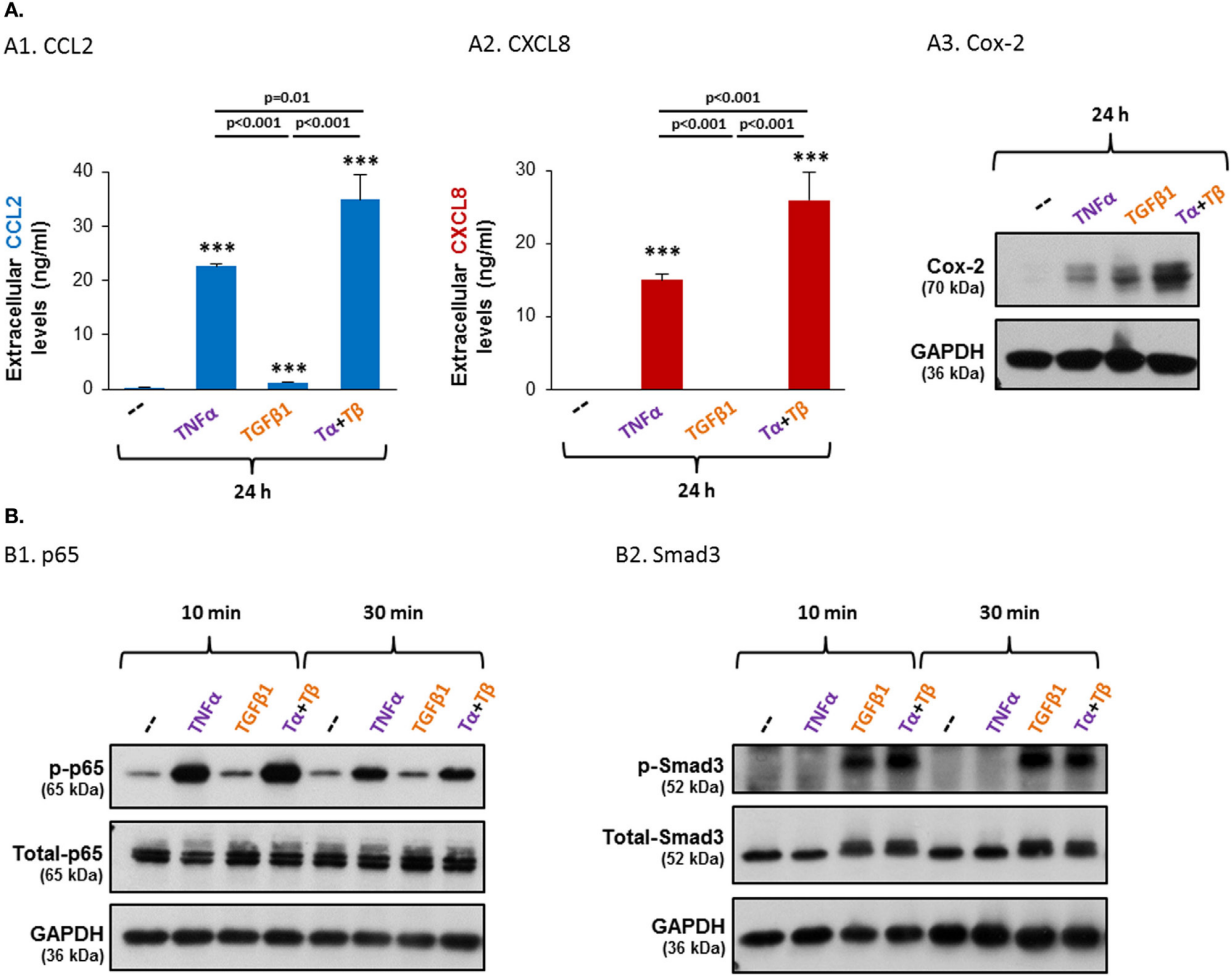
**TNFα and TGFβ1 cooperate in inducing the protein expression levels of CCL2, CXCL8 and Cox-2, and induce the activation of the NF-κB and Smad3 transcription factors, respectively, in MSCs**. Human BM-derived MSCs were stimulated with vehicle (“--”), TNFα (50 ng/ml), TGFβ1 (10 ng/ml), or TNFα + TGFβ1 (same concentrations). Tα + Tβ = TNFα + TGFβ1. **(A)** Protein expression levels of CCL2, CXCL8 and Cox-2. Following 24 h of stimulation (FBS-free medium for CCL2/CXCL8 and 0.5% FBS-containing medium for Cox-2) the extracellular expression of CCL2 [(A1); see also Figure S3 in Supplementary Material] and CXCL8 (A2) was determined by ELISA in cell supernatants, in the linear range of absorbance. ***p ≤ 0.001 compared to vehicle-treated cells. (A3) Cox-2 expression levels were determined by WB; GAPDH was used as a loading control. **(B)** Following 10 and 30 min of stimulation (FBS-free medium), the phosphorylation levels of p65 (B1) and Smad3 (B2) were determined by WB; GAPDH was used as a loading control. In all panels, the findings are representatives of *n* ≥ 3 independent experiments, performed with MSCs of two to three different donors, which have shown similar results.

The above observations, demonstrating that TNFα and TGFβ1 stimulation induced elevated CCL2, CXCL8 and Cox-2 expression in MSCs, and connecting the expression of these pro-malignancy mediators with high expression of TNFα and TGFβ1 in patient samples, have led us to determine the impact of TNFα and TGFβ1 on the protein expression levels of CCL2, CXCL8 and Cox-2 in the MSCs. In line with its strong pro-inflammatory nature, TNFα has potently induced the expression of CCL2 and CXCL8 at the protein level [as we had demonstrated before (41)] and of Cox-2 as well, in the MSCs (Figure 5). TGFβ1 upregulated the mRNA expression of CCL2 in the MSCs but to lower extent than TNFα (Figure 3) and has promoted CCL2 expression to only a small extent at the protein level (Figure 5A1; Figure S3 in Supplementary Material), as could be expected from a cytokine which is not a typical pro-inflammatory mediator. The relatively minor induction of CXCL8 mRNA by TGFβ1—compared to TNFα—did not come into effect at the protein level (Figure 5A2). Yet, TGFβ1 potently induced the protein expression of Cox-2, even to stronger extent than TNFα (Figure 5A3), revealing a potential pro-inflammatory activity for this cytokine.

As TNFα and TGFβ1 are both expressed in MSC-containing niches (as alluded in Section “Introduction”), we next asked what will be the impact of joint stimulation by both TNFα and TGFβ1 together on the MSCs. The findings shown in Figures 5A1–A3 demonstrate cooperativity between the two cytokines, leading to greater effect than their individual impacts. Most importantly, such cooperative effects were evident not only for Cox-2 that was strongly induced by TGFβ1 but also for CCL2 and CXCL8, on which TGFβ1 had a weak or no effect when it was administered alone. These findings indicate that in the presence of TNFα, TGFβ1 acts as a “co-inflammatory” factor that promotes the activities of the classical pro-inflammatory cytokine TNFα. Together, the joint activities of TNFα and TGFβ1 induced a pro-inflammatory phenotype in BM-derived MSCs, demonstrating that factors of the microenvironment can have a strong impact on the fate of MSCs and on the secreted factors they produce.

### The Cooperative Induction of CCL2/CXCL8 by Joint TNFα + TGFβ1 Stimulation Is Differently Regulated by NF-κB and Smad3 than the Cooperative Induction of Cox-2

To identify the molecular mechanisms regulating the joint activities of TNFα + TGFβ1 on MSCs, we analyzed the canonical transcription factors activated by the two cytokines: (1) TNFα activates the NF-κB/p65 pathway (65, 66), which was found in our published findings to regulate TNFα-induced elevation of CCL2 and CXCL8 expression in BM-MSCs (41). The other canonical pathway activated by TNFα, of AP-1, was demonstrated in our past study to be irrelevant in this context (41). (2) The canonical Smad3 pathway that is activated by TGFβ1 (67–69) was investigated in parallel to NF-κB.

Activation analyses indicated that TNFα + TGFβ1 stimulation of MSCs induced prominent phosphorylation of p65 and Smad3 (Figure 5B). p65 was activated by TNFα + TGFβ1 to the same extent as by TNFα alone, and the activation level of Smad3 following TNFα + TGFβ1 stimulation was similar to its activation by TGFβ1 alone. These findings suggested that in the combined TNFα + TGFβ1 stimulation each cytokine activated its respective canonical pathway—TNFα activated p65 and TGFβ1 activated Smad3—and that the functional cooperativity between TNFα + TGFβ1 was due to cooperative activities of p65 and Smad3. Yet, our findings using p65 siRNA and Smad3 siRNA revealed a more complex mode of regulation of TNFα + TGFβ1 activities in the MSCs, as demonstrated further below.

**Figure 6:**
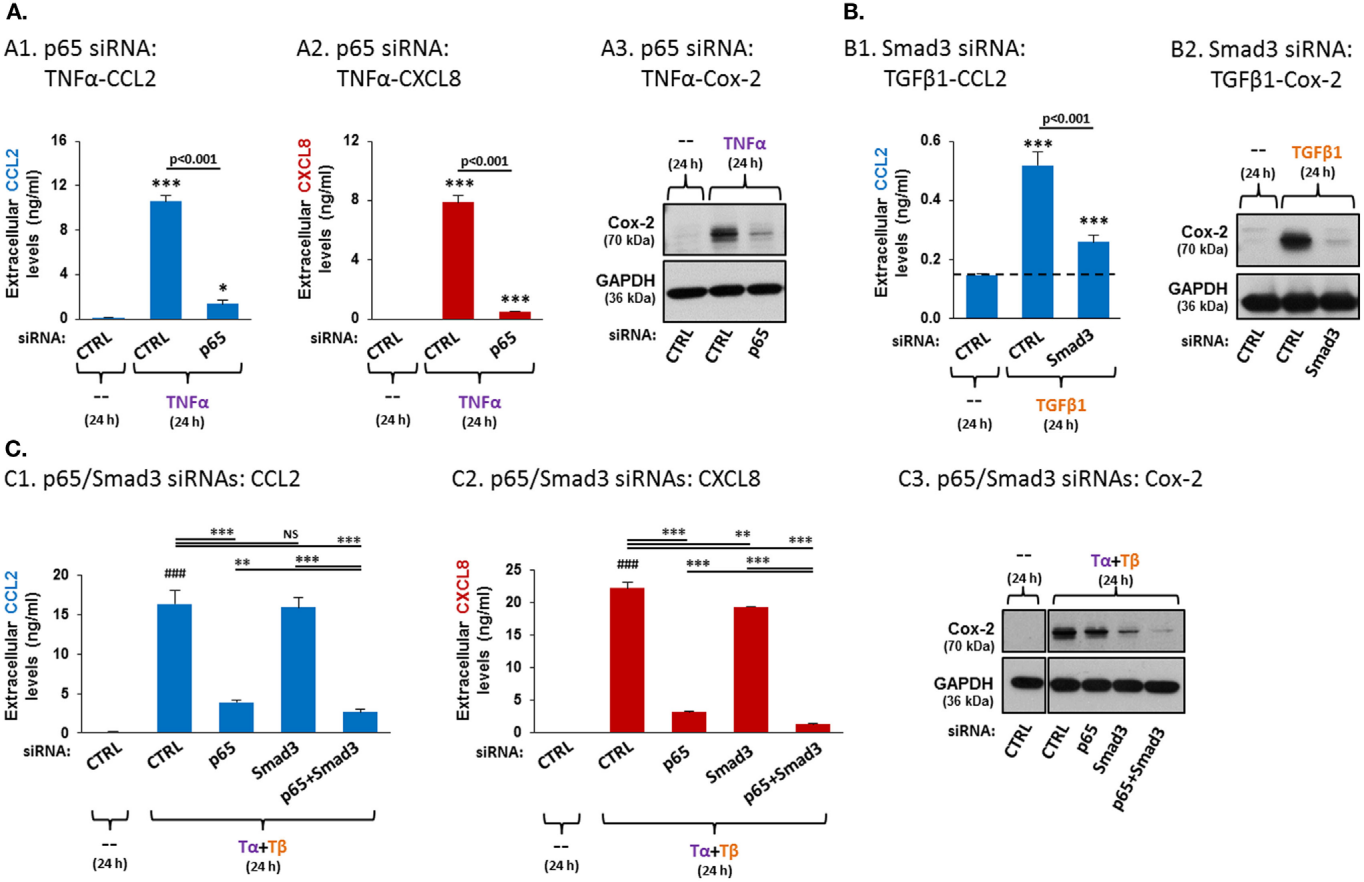
**Following TNFα + TGFβ1 stimulation of MSCs, p65 is the major regulator of CCL2 and CXCL8 expression, while Smad3 is the predominant regulator of Cox-2 expression, cooperating with NF-κB activation**. Human BM-derived MSCs were transiently transfected with control siRNA, with siRNA to p65 or with siRNA to Smad3 and were stimulated as described below. siRNA concentrations were selected based on preliminary titration analysis (data not shown). The expression of CCL2, CXCL8 and Cox-2 was determined as described in Figure 5. **(A)** p65 siRNA effects on TNFα-mediated induction of CCL2 (A1), CXCL8 (A2) and Cox-2 (A3). Following transfection with control siRNA (“CTRL”, 30 nM) or siRNA to p65 (30 nM; efficacy of p65 downregulation is demonstrated in Figure S4A in Supplementary Material), the cells were stimulated with vehicle (“--”) or TNFα (50 ng/ml) for 24 h (0.5% FBS-containing medium). Comment: findings on p65 siRNA effects on TNFα-induced CCL2 and CXCL8 were demonstrated in our published report (41), but in different conditions than in the current study. ***p ≤ 0.001, *p < 0.05 compared to vehicle-treated, control siRNA-transfected cells. **(B)** Smad3 siRNA effects on TGFβ1-mediated induction of CCL2 (B1) and Cox-2 (B2). Following transfection with control siRNA (“CTRL”, 30 nM) or siRNA to Smad3 (30 nM; efficacy of Smad3 down-regulation is demonstrated in Figure S4B in Supplementary Material), the cells were stimulated with vehicle (“--”) or TGFβ1 (10 ng/ml) for 24 h (0.5% FBS-containing medium). ***p ≤ 0.001 compared to vehicle-treated, control siRNA-transfected cells. **(C)** The effects of p65 siRNA and Smad3 siRNA on TNFα + TGFβ1-mediated induction of CCL2 (C1), CXCL8 (C2) and Cox-2 (C3). MSCs were transfected with control siRNA (“CTRL”, 60 nM) or with siRNAs to p65 (30 nM p65 siRNA + 30 nM control siRNA), Smad3 (30 nM Smad3 siRNA + 30 nM control siRNA) or siRNAs to both p65 + Smad3 (30 nM each; efficacies of p65 and Smad3 downregulations are demonstrated in Figures S4C1,C2 in Supplementary Material). Then, the cells were stimulated with vehicle (“--”) or TNFα (50 ng/ml) + TGFβ1 (10 ng/ml) for 24 h (0.5% FBS-containing medium). Tα + Tβ = TNFα + TGFβ1. ***p ≤ 0.001, **p ≤ 0.01. NS, not significant. ^###^*p* ≤ 0.001 compared to vehicle-treated, control siRNA-transfected cells. In all panels, the findings are representatives of *n* = 3 independent experiments, performed with MSCs of two different donors, which have shown similar results. In panel C and in Figures S4C1,C2 in Supplementary Material, all the results were obtained in parallel with MSCs of one donor; similar findings, obtained in MSCs from another donor, are demonstrated in Figure S5 in Supplementary Material.

To determine the roles of NF-κB and Smad3 in TNFα- or TGFβ1-induced increases in CCL2/CXCL8 and Cox-2 we used siRNAs to p65 (NF-κB) and Smad3 (efficacies of p65 and Smad3 downregulation are demonstrated in Figures S4A,B in Supplementary Material, respectively). Under these conditions, the induction of CCL2, CXCL8 and Cox-2 by TNFα alone was markedly dependent on p65 activities (Figure 6A). In parallel, when TGFβ1 acted alone, it promoted the expression of CCL2 and of Cox-2 in a Smad3-dependent mechanism (Figure 6B; CXCL8 was not investigated because it was not affected by TGFβ1 at the protein level, as demonstrated in Figure 5A2).

To test pathway-specificity of TNFα + TGFβ1 effects, we knocked-down both p65 and Smad3 together and then stimulated the MSCs with TNFα + TGFβ1 (efficacies of p65 and Smad3 downregulation are demonstrated in Figures S4C1,C2 in Supplementary Material). The findings of Figure 6C demonstrate that p65 knockdown induced a prominent reduction in the release of CCL2 and CXCL8 from TNFα + TGFβ1-stimulated MSCs, while Smad3 knockdown had no or a very minimal effect and did not add much to p65 downregulation in reducing CCL2 and CXCL8 expression (Figures 6C1,C2; Figure S5 in Supplementary Material demonstrates similar findings in MSCs of another donor—see explanation in Section “Data Presentation and Statistical Analyses”). In contrast, p65 knockdown had only a small effect on the cooperative induction of Cox-2 by TNFα + TGFβ1, but Smad3 downregulation led to substantial reduction in TNFα + TGFβ1-induced Cox-2 expression (Figure 6C3; another donor—Figure S5 in Supplementary Material). The strong elevation in Cox-2 expression following TNFα + TGFβ1 stimulation was almost totally abrogated by joint siRNA-induced downregulation of p65 and Smad3 expression (Figure 6C3; another donor—Figure S5 in Supplementary Material). Overall, our findings demonstrate that the elevated expression of CCL2 and CXCL8 in response to the cooperative activities of TNFα + TGFβ1 was mediated primarily by NF-κB activation, whereas the cooperative induction of Cox-2 by TNFα + TGFβ1 stimulation was mostly dependent on Smad3.

### The Cooperative Induction of CCL2/CXCL8 by Joint TNFα + TGFβ1 Stimulation Is Differently Regulated by TAK1 than the Cooperative Induction of Cox-2

**Figure 7:**
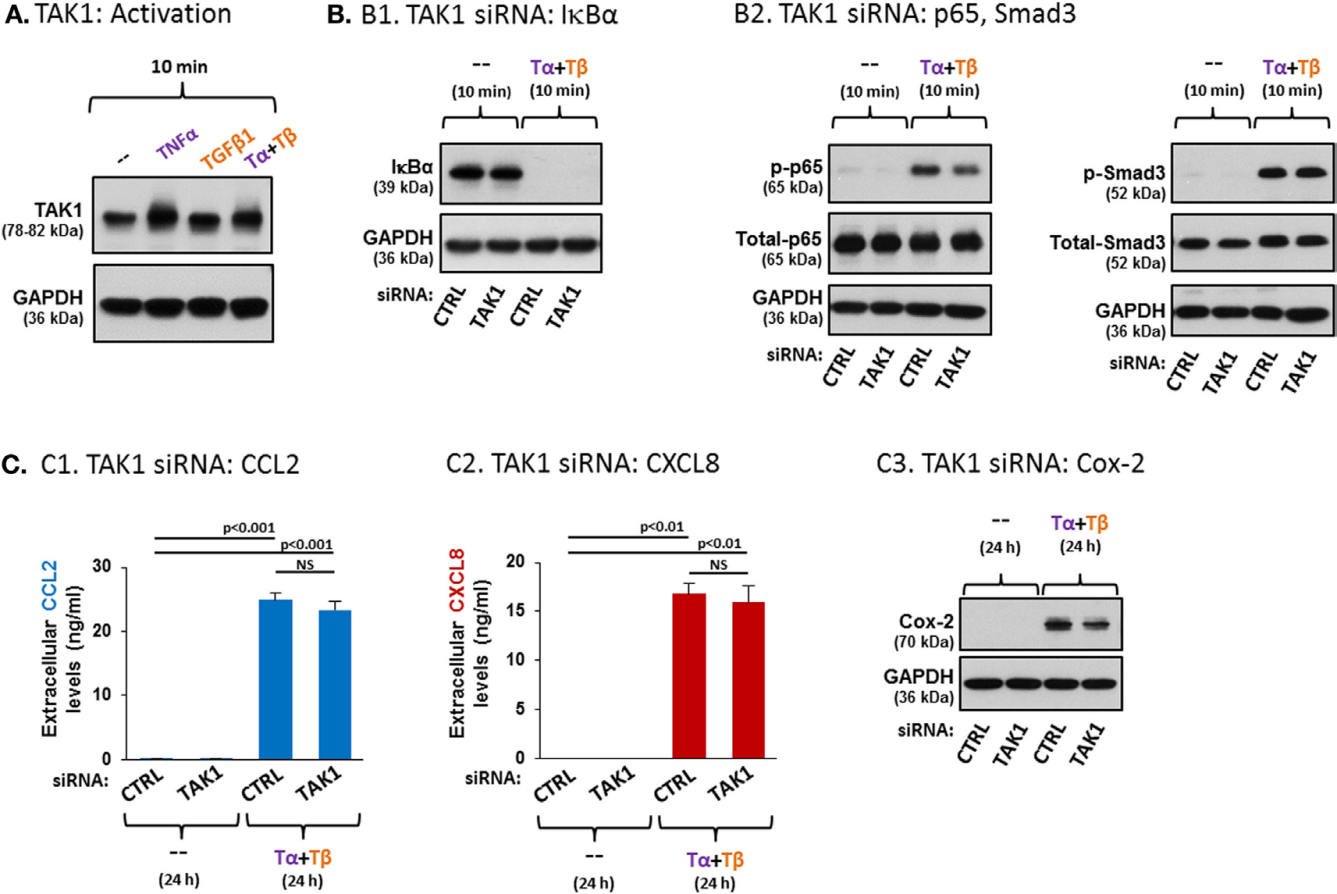
**TAK1 plays divergent roles in regulating TNFα + TGFβ1 cooperatively-induced CCL2/CXCL8 and Cox-2 expression in MSCs**. **(A)** Human BM-derived MSCs were stimulated with vehicle (“--”), TNFα (50 ng/ml), TGFβ1 (10 ng/ml), or TNFα + TGFβ1 (same concentrations) for 10 min (FBS-free medium). Tα + Tβ = TNFα + TGFβ1. TAK1 expression was determined by WB; GAPDH was used as a loading control. The findings are representatives of *n* = 3 independent experiments, performed with MSCs of three different donors, which have shown similar results. **(B,C)** The effects of TAK1 downregulation on MSC signaling and functions. siRNA concentrations were determined by preliminary titration analysis (data not shown). **(B)** Signaling: human BM-derived MSCs were transiently transfected with control siRNA (“CTRL”, 50 nM) or with siRNA to TAK1 (50 nM; efficacy of TAK1 downregulation is demonstrated in Figure S6A1 in Supplementary Material). Following siRNA transfection, the cells were stimulated with vehicle (“--”) or with TNFα (50 ng/ml) + TGFβ1 (10 ng/ml) for 10 min (0.5% FBS-containing medium). Tα + Tβ = TNFα + TGFβ1. (B1) The expression levels of IκBα were determined by WB; GADPH was used as loading control. (B2) p65 and Smad3 activation levels were determined as described in Figure 5. p38 activation levels are demonstrated in Figure S6A2 in Supplementary Material. **(C)** Function: human BM-derived MSCs were transiently transfected with control siRNA (“CTRL”, 50 nM) or with siRNA to TAK1 (50 nM; efficacies of TAK1 downregulation are demonstrated in Figures S6B1,B2 in Supplementary Material). Following siRNA transfection, the cells were stimulated with vehicle (“--”) or with TNFα (50 ng/ml) + TGFβ1 (10 ng/ml) for 24 h (0.5% FBS-containing medium). Tα + Tβ = TNFα + TGFβ1. The impact of TAK1 siRNA on the expression of CCL2 (C1), CXCL8 (C2) and Cox-2 (C3) was determined as described in Figure 5. NS, not significant. In all panels, the findings are representatives of *n* = 3 independent experiments, performed with MSCs of two different donors, which have shown similar results. Similar findings, obtained in MSCs from another donor, are demonstrated in Figure S7 in Supplementary Material (each panel with its corresponding validation of downregulation efficacy).

In parallel to the canonical pathways activated by TNFα and TGFβ1, the two cytokines share the ability to activate the pathway of the MAP3K named TAK1 (69–72). Previous studies have indicated that TAK1 activation leads to activation of NF-κB, but does not induce direct activation of Smad3. Activated TAK1 undergoes posttranslational modifications including K63-linked activating ubiquitination as well as phosphorylation at serine/threonine residues (70, 73–77), which are difficult to detect at endogenous levels with existing experimental tools (data not shown). These activation-associated posttranslational modifications of TAK1 can be reflected by a smeared gel mobility shift (75–77). Accordingly, we found that stimulation of the MSCs by TNFα, alone or in the presence of TGFβ1, had induced a smeared migration shift in TAK1 (Figure 7A); no such migration shift was detected following TGFβ1 stimulation, suggesting that TGFβ1 did not activate TAK1 in the MSCs.

To determine the roles of TAK1 in the ability of TNFα + TGFβ1 to cooperatively induce the pro-inflammatory factors, siRNA to TAK1 was used, demonstrating high efficiency in downregulating TAK1 expression [Figure S6 in Supplementary Material; the common inhibitor of TAK1, 5Z-7-oxozeaenol, was not used due to recent reports raising concerns on its specificity (78, 79)]. Published reports indicate that downstream of TAK1 activation, IKK is activated and leads to phosphorylation of IκBα; upon phosphorylation, this negative regulator of NF-κB is destined for degradation, thus enabling the activation of p65 (80). Therefore, in this part of the study, we first asked what is the influence of TAK1 siRNA on IκBα expression levels following costimulation of the MSCs by TNFα + TGFβ1. Our findings indicate that following MSC stimulation by TNFα + TGFβ1, IκBα was diminished in the cells (Figure 7B1; another donor—Figure S7 in Supplementary Material). This finding agrees with our previous results showing that this combined stimulation induces NF-κB activation (Figure 5B1). Yet, siRNA to TAK1 did not affect IκBα degradation following TNFα + TGFβ1 stimulation, suggesting that TAK1 is only minimally involved in the regulation of the NF-κB pathway in TNFα + TGFβ1-stimulated MSCs, if at all (Figure 7B1; another donor—Figure S7 in Supplementary Material). Accordingly, when we determined the effects of siRNA to TAK1 on the activation of p65 or of p38, which is another typical downstream target of TAK1 (73, 81, 82), we found out that TAK1 downregulation by siRNA led to only minor reduction in the activation of p65 (Figure 7B2; another donor—Figure S7A3 in Supplementary Material) or of p38 (Figure S6A2 in Supplementary Material; another donor—Figure S7A4 in Supplementary Material). As expected, TAK1 did not regulate Smad3 activation in MSCs (Figure 7B2; another donor—Figure S7A4 in Supplementary Material).

In view of the strong involvement of p65 in the cooperative induction of CCL2 and CXCL8 by TNFα + TGFβ1 stimulation (Figures 6C1,C2) and of the minimal reduction in p65 activation following TAK1 downregulation (Figure 7B2; Figure S7A3 in Supplementary Material), it was not surprising that TAK1 knockdown (efficacies—Figure S6B1 in Supplementary Material) did not impact the expression of CCL2 and CXCL8 (Figures 7C1,C2; another donor—Figure S7B in Supplementary Material). However, TAK1 downregulation did lead to reduced production of Cox-2 following TNFα + TGFβ1 stimulation of the MSCs (Figure 7C3; another donor—Figure S7C in Supplementary Material). These latter findings agree with the relatively lower involvement of p65 in TNFα + TGFβ1-induced Cox-2 expression, compared to TNFα + TGFβ1-induced CCL2 and CXCL8 expression in the MSCs (Figure 6C). Thus, our findings reveal divergent roles for TAK1 in regulating the expression of different TNFα + TGFβ1-induced pro-inflammatory mediators in MSCs.

### TNFα + TGFβ1-Stimulated MSCs Release Factors That Promote Elongation, Migration, and Scattering of Breast Tumor Cells

TNFα and TGFβ1 are both expressed in many tumors (20–26) and, as our findings so far indicate, act in cooperativity to promote the pro-inflammatory phenotype of MSCs, which also prevail in breast tumors (20–23). Moreover, signaling in MSCs *via* TNFα and TGFβ1 has been strongly associated with tumor progression (20–23). To follow on the above-mentioned observations, we were interested in identifying the combined impact of TNFα and TGFβ1 on the generation by MSCs of factors that may contribute to increased tumor cell motility. To this end, we determined the effects of factors that were secreted by MSCs—following their activation by TNFα + TGFβ1 together—on characteristics of breast tumor cells that are connected to increased motility and spreading. To specifically isolate the effects delivered by factors released by the stimulated MSCs from signals that may be induced in the tumor cells by the cytokines themselves [as shown in Ref. (24, 83–90)], a control group consisting of TNFα + TGFβ1 stimulation (without factors of the MSCs) was included in the analyses.

In this part of the study, two human breast tumor cells were addressed: the highly motile human MDA-MB-231 triple negative cells and the relatively less invasive MCF-7 luminal-A human breast tumor cells (91, 92). Following stimulation of the MSCs for 24 h by TNFα + TGFβ1 or by their vehicles, the CM of cytokine-stimulated or of vehicle-exposed cells (Groups 4 and 3, respectively) were transferred to mCherry-expressing breast tumor cells. Control tumor cells were grown in parallel with medium alone (Group 1) or with TNFα + TGFβ1 only (Group 2).

**Figure 8:**
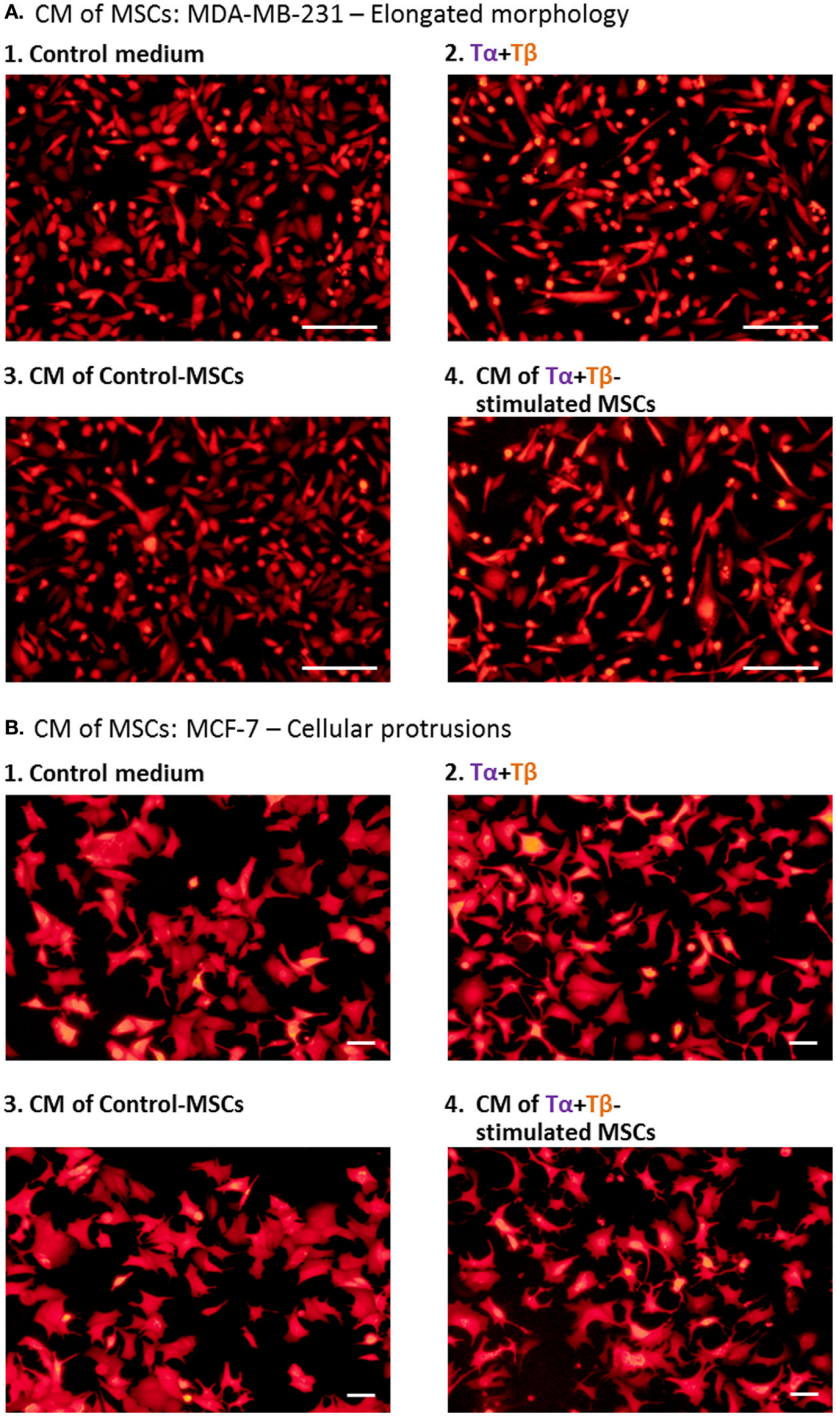
**Factors released by TNFα + TGFβ1-stimulated MSCs induce cellular elongation and formation of cellular protrusions in human breast cancer cells**. Human BM-derived MSCs were stimulated with vehicle (“Control-MSCs”) or TNFα (50 ng/ml) + TGFβ1 (10 ng/ml), in 0.5% FBS-containing medium; Tα + Tβ = TNFα + TGFβ1. In parallel, samples of “Control medium” (not exposed to MSCs), with or without the stimulating cytokines, were kept in identical conditions. Twenty-four hours later, all different media were filtered (0.45 µm pores) and applied to mCherry-expressing MDA-MB-231 cells for 48–72 h, in different experiments **(A)** or to mCherry-expressing MCF-7 cells for 48 h **(B)**. Cancer cells were then washed and photographed. CM = conditioned media. Scale bar: 200 µm in MDA-MB-231 cells and 50 µm in MCF-7 cells. The pictures are representatives of *n* ≥ 3 independent experiments, performed with MSCs of two to three different donors, which have shown similar results. For MDA-MB-231 cells, enlarged pictures of cells, obtained in MSCs from another experiment, are demonstrated in Figure S8 in Supplementary Material.

In view of the high basal motility of MDA-MB-231 cells, we chose to determine the impact of the different CM on the generation of elongated morphology of these cells. This has been connected to elevated tumor cell motility (93, 94). The results of Figure 8A indicate that CM derived from TNFα + TGFβ1-stimulated MSCs (Group 4) have induced an elongated morphology in the tumor cells. The results indicated that the influence of the CM derived from TNFα + TGFβ1-stimulated MSCs (Group 4) was much stronger than the effects induced on the tumor cells by the CM obtained from vehicle-treated MSCs (Group 3) and was also more evident than the impact of the cytokines themselves (Group 2; photos demonstrating enlarged cell magnifications of MDA-MB-231 in another experiment are demonstrated in Figure S8 in Supplementary Material). These findings indicate that factors released by TNFα + TGFβ1-stimulated MSCs enhanced a malignancy-related characteristic in the tumor cells.

**Figure 9:**
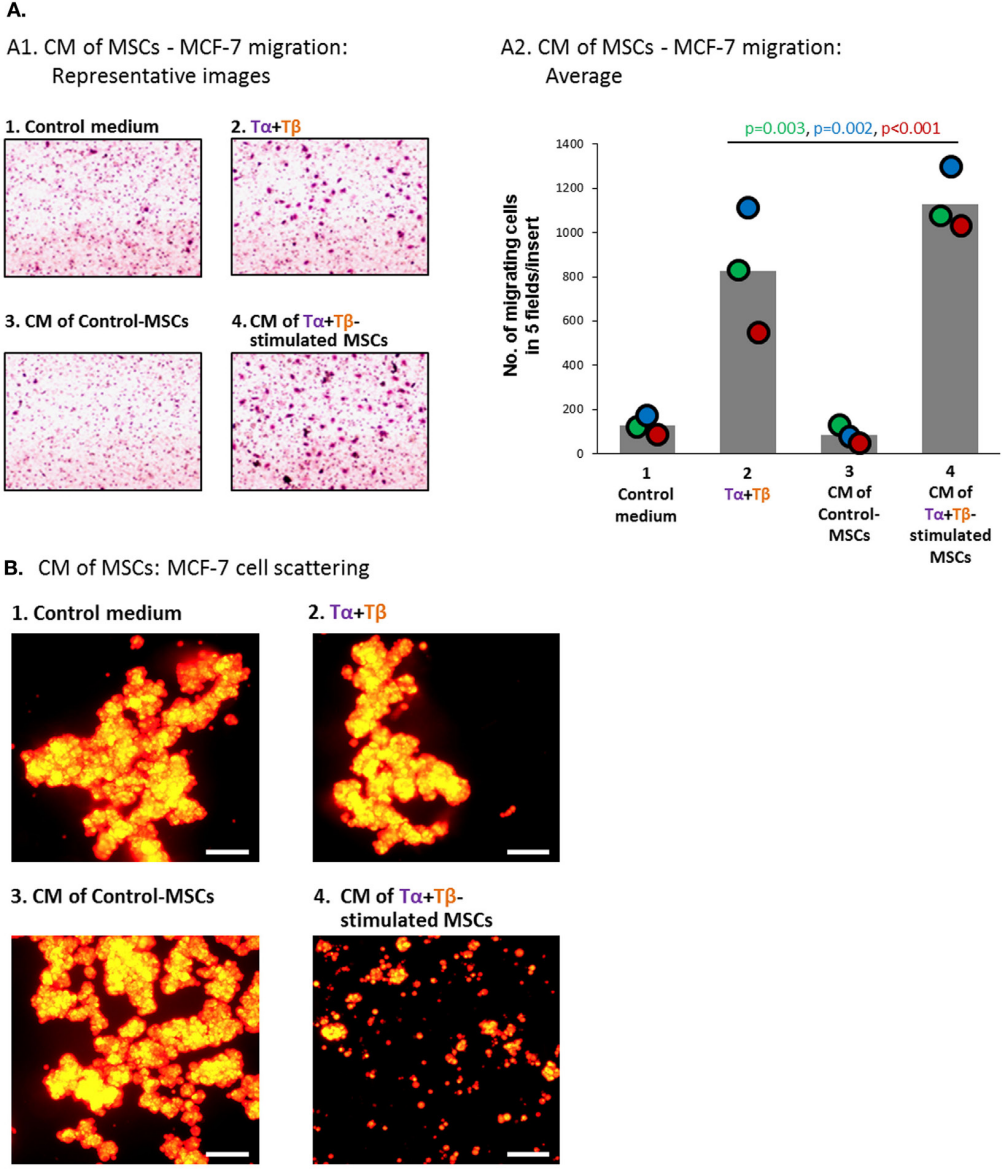
**Factors released by TNFα + TGFβ1-stimulated MSCs induce elevated migration and scattering of MCF-7 breast cancer cells**. Human BM-derived MSCs were stimulated with vehicle (“Control-MSCs”) or TNFα (50 ng/ml) + TGFβ1 (10 ng/ml) [0.5% FBS-containing medium in panel **(A)** and FBS-free medium in panel **(B)**]; Tα + Tβ = TNFα + TGFβ1. In parallel, samples of “Control medium” (not exposed to MSCs), with or without the stimulating cytokines, were kept in identical conditions. Twenty-four hours later, all different media were filtered (0.45 µm pores) and applied to mCherry-expressing MCF-7 cells for 48 h **(A)** or 96 h **(B)**. Then, functional assays were performed. CM = conditioned media. **(A)** Migration of MCF-7 cells toward 10% FBS-containing medium. (A1) Representative pictures of part of the high-resolution fields, ×40 magnification, of one of three independent experiments performed with MSCs of two different donors. (A2) Bar graph demonstrating the average number of cells migrating in each cell group, obtained in three independent experiments (total of five pictures/insert in each experiment); colored dots represent the number of migrating cells in each of these same three experiments, with corresponding color-coded *p*-values indicated. **(B)** Scattering of MCF-7 cells out of tumor spheroids. Scale bar = 200 µm. The pictures are representatives of *n* > 3 independent experiments, performed with MSCs of three different donors that have shown similar results.

In parallel, the relatively low basal motility phenotype of MCF-7 cells has motivated us to ask if factors released by TNFα + TGFβ1-stimulated MSCs would increase the formation of cellular protrusions and motility in MCF-7 cells. Here, we found that the CM of TNFα + TGFβ1-stimulated MSCs (Group 4) have led to formation of definite cellular protrusions that were generally more intense than in the relevant control groups (Figure 8B); such protrusions were strongly connected in other studies to increased EMT and scattering of these cells (86, 95). Moreover, when we determined the motility of MCF-7 cells in response to migration-inducing factors that are present in serum, we found that cancer cells cultured in the presence of TNFα + TGFβ1-derived CM (Group 4) revealed more robust migratory ability than tumor cells exposed to the other treatments, including CM derived from vehicle-treated MSCs (Group 3) or the cytokines alone (Group 2; Figures 9A1,A2).

To further investigate the impact of factors released by TNFα + TGFβ1-stimulated MSCs on tumor cell motility, we determined the scattering of MCF-7 cells from spheroid tumor masses, demonstrated by our published study to correlate with a more aggressive behavior of the cells (86). Using a 3D spheroid-based detachment assay, we found that MCF-7 cells treated by CM of TNFα + TGFβ1-stimulated MSCs (Group 4) had very high capabilities of scattering out of the 3D tumor spheroids (Figure 9B). A remarkable difference was revealed between MCF-7 cells exposed to CM derived from TNFα + TGFβ1-stimulated MSCs (Group 4) and cancer cells exposed to the two other relevant treatments: CM of vehicle-treated MSCs (Group 3) or to the two cytokines only (Group 2).

All of the above findings demonstrate the high ability of CM of TNFα + TGFβ1-stimulated MSCs (Group 4) to promote motility-related functions of breast tumor cells *in vitro*; importantly, the effects of CM derived from TNFα + TGFβ1-stimulated MSCs were more pronounced than the effects of CM derived from vehicle-treated MSCs (Group 3) or of the cytokines alone (Group 2). These findings clearly indicate that factors produced by MSCs following TNFα + TGFβ1 stimulation induced a motile phenotype in cancer cells, which can potentially contribute to elevated aggressiveness.

## Discussion

Mesenchymal stem cells, their functions and modes of regulation, have been extensively studied during the last several years. The growing interest in these cells stems from their ability to give rise to cartilage, bone, muscle, and fat lineages (1–3); their activities in sites of hematopoiesis and inflammation (4, 5); their prominent roles in controlling malignancy (96–98); and their potential use as tools for gene delivery and tissue regeneration (7, 8). Stemming out from different tissue origins, the high plasticity of MSCs and their abundance at many organs emphasize the need to identify how their functions are regulated by factors of their intimate microenvironments.

Accordingly, in this study we were interested in deciphering the combined effects of TNFα + TGFβ1, both being coexpressed in different niches and conditions (16, 17, 20–23), on MSCs. Although TNFα and TGFβ1 very often have opposing roles in immune/inflammatory regulation (9–13), we found out that they join forces and act in cooperativity to promote the pro-inflammatory phenotype of MSCs. In the presence of TNFα, TGFβ1 turned into a co-inflammatory cytokine whose functions promoted the activities mediated by the classic pro-inflammatory cytokine TNFα. These findings add to recent reports on the ability of TGFβ to promote immune responses by reversing the suppressive activity of MSCs on T cell proliferation (99).

The findings obtained in our study reveal that the functional cooperativity between TNFα + TGFβ1 leads to activation of NF-κB and Smad3, yet the involvement of the two transcription factors in regulating the pro-inflammatory phenotype of the MSCs is complex. When acting alone, TNFα-induced expression of CCL2, CXCL8 and Cox-2 depended on NF-κB activation, agreeing with published reports on direct binding of p65 to the promoter/enhancer regions of these genes [e.g., Ref. (100–102)]. In parallel, when the MSCs were stimulated only by TGFβ1, the induction of CCL2 and Cox-2 highly depended on Smad3 activities, reflecting the presence of Smad3-binding sites in these two genes (103, 104). Yet, the cooperative induction of CCL2, CXCL8 and Cox-2 by joint stimulation with TNFα + TGFβ1 was due to modified balance between NF-κB and Smad3.

Specifically, NF-κB activation was almost exclusively involved in TNFα + TGFβ1-induced CCL2 and CXCL8 without a significant involvement of Smad3. In contrast, the cooperative activities of TNFα + TGFβ1 leading to elevated expression of Cox-2 were mostly dependent on Smad3 activation. These findings may reflect the importance of cooperativity between different transcription factors regulating the concerted transcription of different pro-inflammatory targets in MSCs, as has been reported in other systems [e.g., Ref. (100, 105)]. Most evidently, this is the case for the TGFβ1-Smad3 pathway: the wide variety of TGFβ1 activities reflect the physical interactions of Smad3 with different transcription factors, leading to one response when interacting with one specific transcription factor while generating another response upon interaction with a different transcription factor (106, 107). Indeed, this complex mode of TGFβ1-induced Smad3 activation may stand in the basis of joint Smad3 and p65 activities in TNFα + TGFβ1-stimulated MSCs, when the Cox-2 response was induced. Cooperativity between different transcription factors may also be involved in TNFα + TGFβ1-induced elevation in CCL2 and CXCL8 expression: the phosphorylation levels of p65 upon TNFα + TGFβ1 stimulation were not higher than with TNFα alone, proposing that TGFβ1 stimulation has not changed the activation level of p65 but rather modified the cooperativity between p65 and other transcription factors that act together with it on the relevant genes.

Our findings on the roles of TAK1 reveal additional aspects of differential regulation of CCL2/CXCL8 compared to Cox-2. It is interesting to note that unlike other cell systems (81, 108), TAK1 was not a key regulator of NF-κB activation in the MSCs; it also did not regulate the expression of CCL2 and CXCL8 while partly controlling the expression of Cox-2. The roles of TAK1 in this system nicely reflect the fact that different targets of TNFα + TGFβ1 are regulated in a divergent manner by NF-κB activation, being almost exclusively involved in CCL2/CXCL8 induction and only partly active in Cox-2 induction upon TNFα + TGFβ1 stimulation.

Thus, in the current study we identify molecular mechanisms induced by joint activities of different factors that are coexpressed at specific niches/conditions. Cancer is a major clinical implication in which the joint activities of TNFα + TGFβ1 on MSCs are very relevant. It has been demonstrated that the two cytokines cooperate in driving epithelial-to-mesenchymal processes and the generation of cancer stem cells in breast cancer, colorectal cancer, and other malignancies (24–26, 43). Our findings propose that when the TME is enriched with both cytokines, as may often be the case in malignancy (20–26), TNFα + TGFβ1 would act not only on the tumor cells but also may induce the release of pro-inflammatory and tumor-promoting factors by MSCs. The factors released by the MSCs, which are cells with well-established tumor-promoting roles (109–113), can contribute to cancer progression by promoting two complementing processes: (1) they may enrich the TME with pro-inflammatory mediators that have been identified as major contributors to tumor progression, such as CCL2, CXCL8 and Cox-2 (37–39, 62, 63, 114); (2) In parallel, the factors released by the TNFα + TGFβ1-stimulated MSCs, pro-inflammatory and others, may act directly on the cancer cells to promote their migratory and invasive properties, as we have shown in the current study.

Therefore, on the whole, the presence of both TNFα and TGFβ1 at tumor sites, combined with the factors they induce in MSCs (as we have demonstrated), may have pro-malignancy effects that act on the TME as well as directly on the cancer cells, to promote their pro-invasive potential. Along these lines, in recent preliminary studies, we have generated mRNA expression profiles of breast tumor cells grown in the presence of CM derived from TNFα + TGFβ1-stimulated MSCs. In this ongoing study, we identified that such CM has led to elevated expression in the tumor cells of molecules that control the organization of the actin cytoskeleton and of microtubules, and promote migration/invasion, matrix degradation, and metastasis in breast cancer: Rho GTPase 1, laminin gamma 2, LIM-only protein FHL2, MMP9, tubulin β3, ICAM-1, MMP13, zyxin, WASP interacting protein, and myosin X. Thus, it is expected that in future studies we will be able to identify the molecules that drive the pro-migratory phenotype of breast tumor cells following their exposure to factors released by TNFα + TGFβ1-stimulated MSCs.

Our findings demonstrating the joint power of TNFα + TGFβ1 + the factors they induce in MSCs on cancer cells provide proof-of-concept to the fact that MSCs are strongly affected by their microenvironment, and as a result secrete soluble mediators that modify their surroundings. These observations are of high relevance to different physiological and pathological settings in which the two cytokines are coexpressed, alongside with MSCs. First and outmost, the pro-inflammatory phenotype gained by TNFα + TGFβ1-stimulated MSCs is very relevant to immune regulation, where MSCs are playing important roles. MSCs are generally considered as having one of two phenotypes: (1) “pro-inflammatory” MSC1 cells: in microenvironment low in inflammatory signals, these cells polarize to a pro-inflammatory phenotype, inducing the generation of activated T cells; and (2) “anti-inflammatory” MSC2 cells: when the microenvironment is enriched with pro-inflammatory mediators, MSC2 cells turn into the anti-inflammatory/immunosuppressive type (115, 116). Our findings raise questions on the way these two subpopulations of MSCs would act when exposed to both TNFα + TGFβ1, because in contrast to the general view seeing them as having opposing forces in immune regulation—pro-inflammatory *vs*. anti-inflammatory/immunosuppressive, respectively—we demonstrate in this study that the MSCs gained an enhanced pro-inflammatory phenotype when stimulated jointly by TNFα + TGFβ1. Obviously, this issue deserves in-depth investigation of its own; yet, it is worth mentioning some relevant findings from our current study: although the mRNA array analysis of our study indicated that many pro-inflammatory mediators are induced by the cytokines, it has demonstrated that TNFα and TGFβ1 did not modify the expression levels of immune molecules associated with immune suppression, including IDO1, IDO2, CTLA-4, and PD-L1.

Much beyond regulation of immune activities, many other health-related conditions may be affected by MSC exposure to both TNFα and TGFβ1 together. Our current understanding of fracture healing suggests that in this setting both cytokines are necessary for inducing MSC migration and/or MSC activities that are required for fracture healing (16, 17). As a result of pro-inflammatory processes that are ensued by TNFα, macrophages that are recruited to bones release chemokines such as CCL2, which promote MSC recruitment and function. In parallel, macrophages release TGFβ1 that promotes the proliferation and differentiation of MSCs, thus enhancing processes of bone repair (16, 17). However, the delicate equilibrium between these factors may be impaired in aging individuals by extensive pro-inflammatory processes, whose trigger(s) are not fully identified (6, 18, 19). Our findings raise the possibility that such increased pro-inflammatory phenotype may be gained by the MSCs due to their exposure to TNFα and TGFβ1 simultaneously, at the bone niche.

The relevance of cooperative TNFα + TGFβ1 activities on MSCs can be further extended to pathological conditions in which the two cytokines are coexpressed, alongside with the presence of MSCs, or in which MSC-based therapies are considered. These include pulmonary diseases (8, 117), cardiomyopathy (118–120), and possibly also Alzheimer’s disease (121, 122). In these conditions, it is possible that the combined activities of TNFα + TGFβ1 promote—through their impact on MSCs—the pro-inflammatory nature of the microenvironment.

Moreover, such mechanisms may be particularly relevant when MSCs are considered as therapeutic tools. Gene-modified MSCs which are delivered from exogenous sources may be affected by the intimate microenvironment residing in their respective niche, which may be enriched with both TNFα and TGFβ1. In response to the two cytokines, such MSCs may turn into pro-inflammatory reservoirs, acting in unbalanced and undesired manners and affecting the functions of other cells in their vicinity. If so, it would be desired to inhibit the joint activities of TNFα and TGFβ1 by targeting their receptors or downstream mediators. The molecular complexity revealed in our study suggests that combined targeting of multiple pathways may be required. Eventually, it is the intricate nature of molecular pathways driven by different microenvironment stimuli that will dictate the therapeutic measures that are needed in each setting.

To follow up on our observations, it is important to note that not only cooperativity may be taking place between TNFα + TGFβ1 as we have demonstrated but also other modes of cross talks between them may take place as well. A cascade-type of interaction was found in adipose tissue-derived MSCs, which upon priming with TNFα released TGFβ1 that, in turn, elevated the malignancy phenotype of breast tumor cells (27). Here, it is interesting to note that in our studies TNFα did not induce an elevated expression of TGFβ1 mRNA, and TGFβ1 did not increase TNFα expression (as indicated in the mRNA array). Thus, it is possible that regulation of MSCs depends not only on the content of microenvironmental factors but also on the tissue origin of the MSCs.

Overall, our findings indicate that the microenvironment has a strong impact on the phenotype of MSCs and their functions. The content of the microenvironment and the origin of MSCs may be critical factors in driving molecular processes that eventually affect tissue cells in their vicinity. These findings have strong implications not only on MSCs that are found natively at different niches but also on therapeutic modalities that are based on MSCs delivered to different tissues from external sources.

## Author Contributions

SL was responsible for gathering most of the experimental data and their analysis and had instrumental contribution to study design, data interpretation, and drafting the manuscript; YL contributed to data gathering throughout the whole study and supported additional aspects of the project; AB participated in design and data gathering of mRNA arrays and the validation of mRNA expression by qPCR; KA has performed bioinformatics analyses of mRNA array data; NO participated in WB analyses; AY participated in analysis of mRNA array data; CK performed the analyses of METABRIC and TCGA datasets; TM has supported the qPCR analyses; SW participated in study conception and design; AB-B was the principal investigator responsible for study conception and design and for complete manuscript preparation.

## Conflict of Interest Statement

The authors declare that the research was conducted in the absence of any commercial or financial relationships that could be construed as a potential conflict of interest.
