# Supplementary material for: Co-Inflammatory Roles of TGFβ1 in the Presence of TNFα Drive a Pro-inflammatory Fate in Mesenchymal Stem Cells
Source: Front Immunol. 2017 May 11;8:479. doi: 10.3389/fimmu.2017.00479 (PMC5425596; doi:10.3389/fimmu.2017.00479)
Supplement: Supplementary file 1 [file Data_Sheet_1.pdf]

Supplementary Materials

Co-inflammatory roles of TGFβ1 in the presence of TNFα drive a pro-inflammatory fate in mesenchymal stem cells

S. Lerrer, Y. Liubomirski, A. Bott, K. Abnaof, N. Oren, A. Yousaf, C. Körner, T. Meshel, S. Wiemann, A. Ben-Baruch

Corresponding author
Adit Ben-Baruch
aditbb@tauex.tau.ac.il

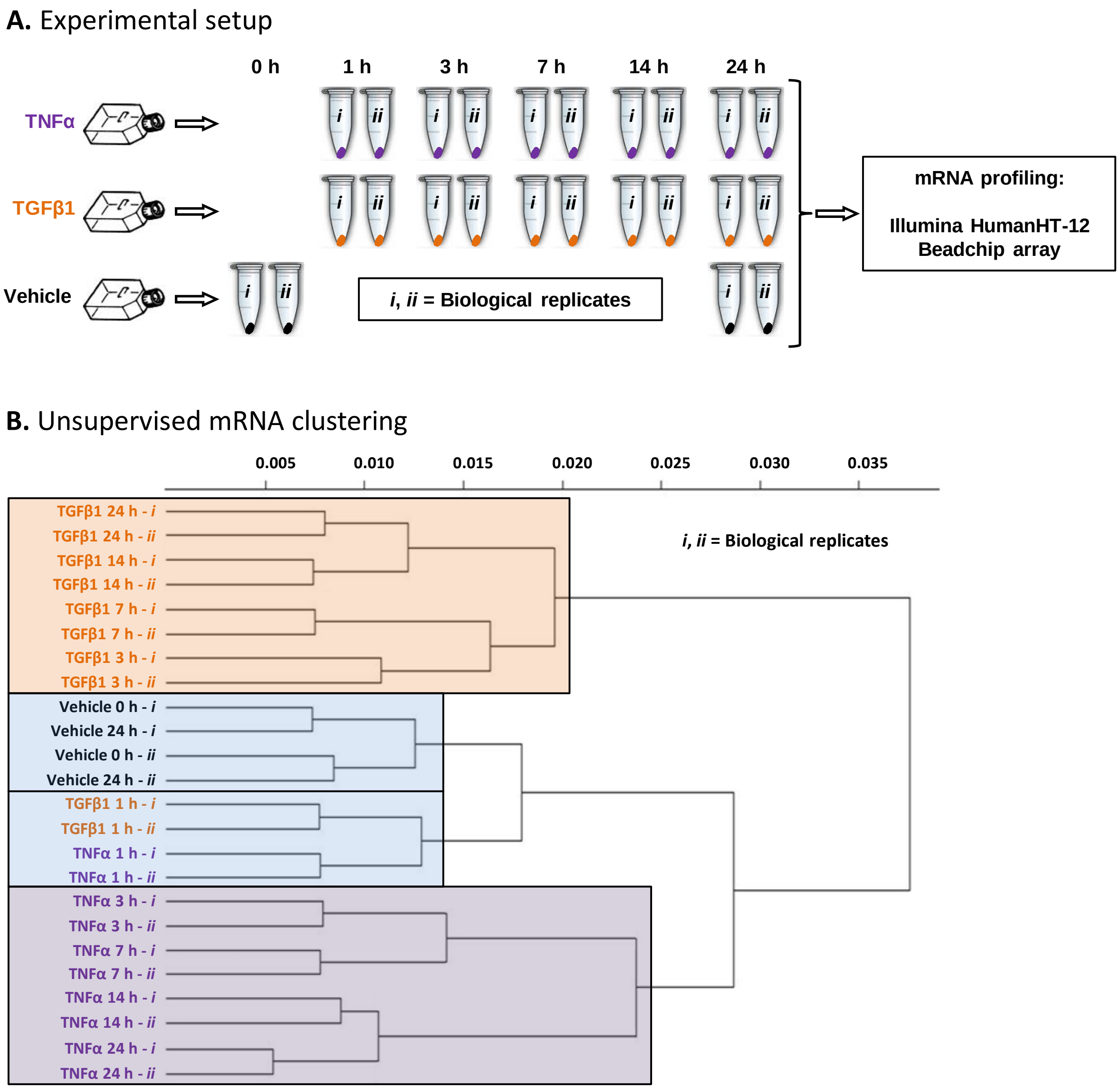

<u>**Supplementary Figure 1**</u>

Experimental setup of MSC stimulation by TNFα and TGFβ1 for mRNA arrays, and data of unsupervised clustering

Human BM-derived MSCs were stimulated with TNFα (50ng/ml) or TGFβ1 (10ng/ml), or treated by a vehicle control. Cytokine concentrations were selected as described in "Materials and methods". **(A)** For mRNA arrays, MSCs of Donor #1 were stimulated by the cytokines for the indicated time points, in two separate biological repeats ("*i*, *ii*"). qPCR validations of 18 up-regulated mRNAs were performed in MSCs of Donors #2 and #3, as demonstrated in Figure 3. **(B)** Unsupervised clustering of total mRNAs included in the array.

METABRIC: Target correlations

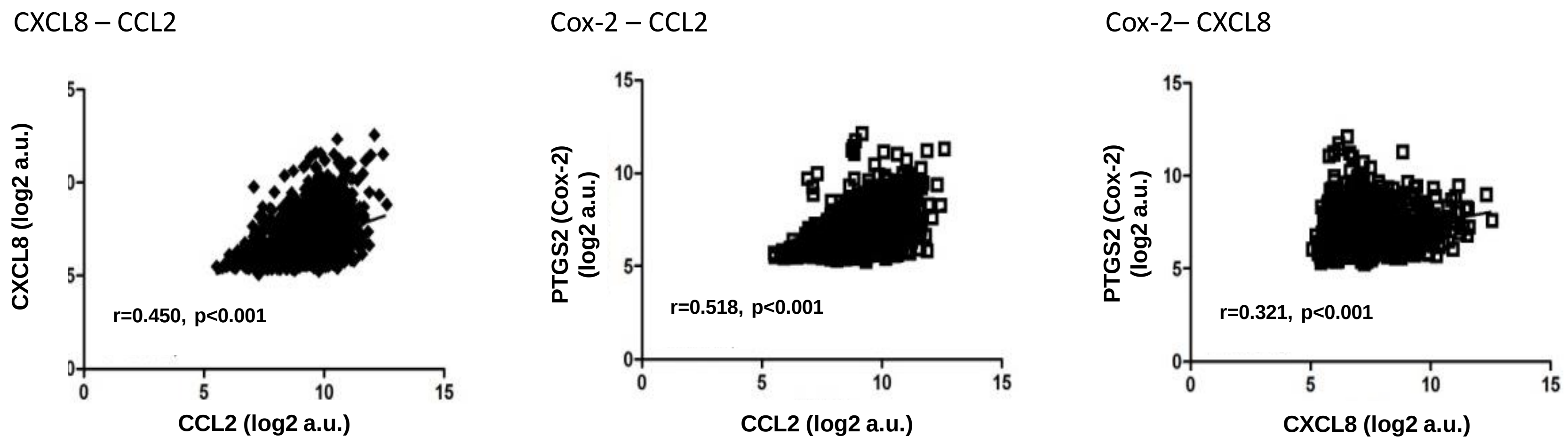

Supplementary Figure 2

Coordinated expression of CCL2, CXCL8 and Cox-2 in patient samples of the METABRIC dataset

The METABRIC dataset, consisting of 1992 samples of breast cancer patients, was used to determine the correlations between CCL2, CXCL8 and PTGS2 (Cox-2) expression levels. a.u, Arbitrary Units. Similar analysis performed with the TCGA dataset of patient samples is demonstrated in Figure 4. Details on statistical analysis are provided in Figure 4B.

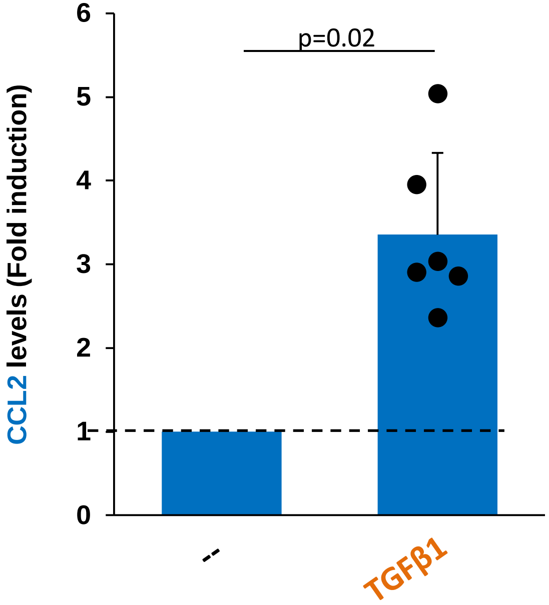

<u>Supplementary Figure 3</u>

TGFβ1 stimulation elevates the protein expression levels of CCL2 in MSCs

Human BM-derived MSCs were stimulated with TGFβ1 (10ng/ml) for 24 hours, or treated by a vehicle control (--). CCL2 levels in cell supernatants were determined by ELISA, at the linear range of absorbance. The figure demonstrates fold change of CCL2 release, independently determined in MSCs of 6 different donors. The bar represents mean ± SD and each dot represents the fold change value obtained in one donor out of the 6 different MSCs donors.

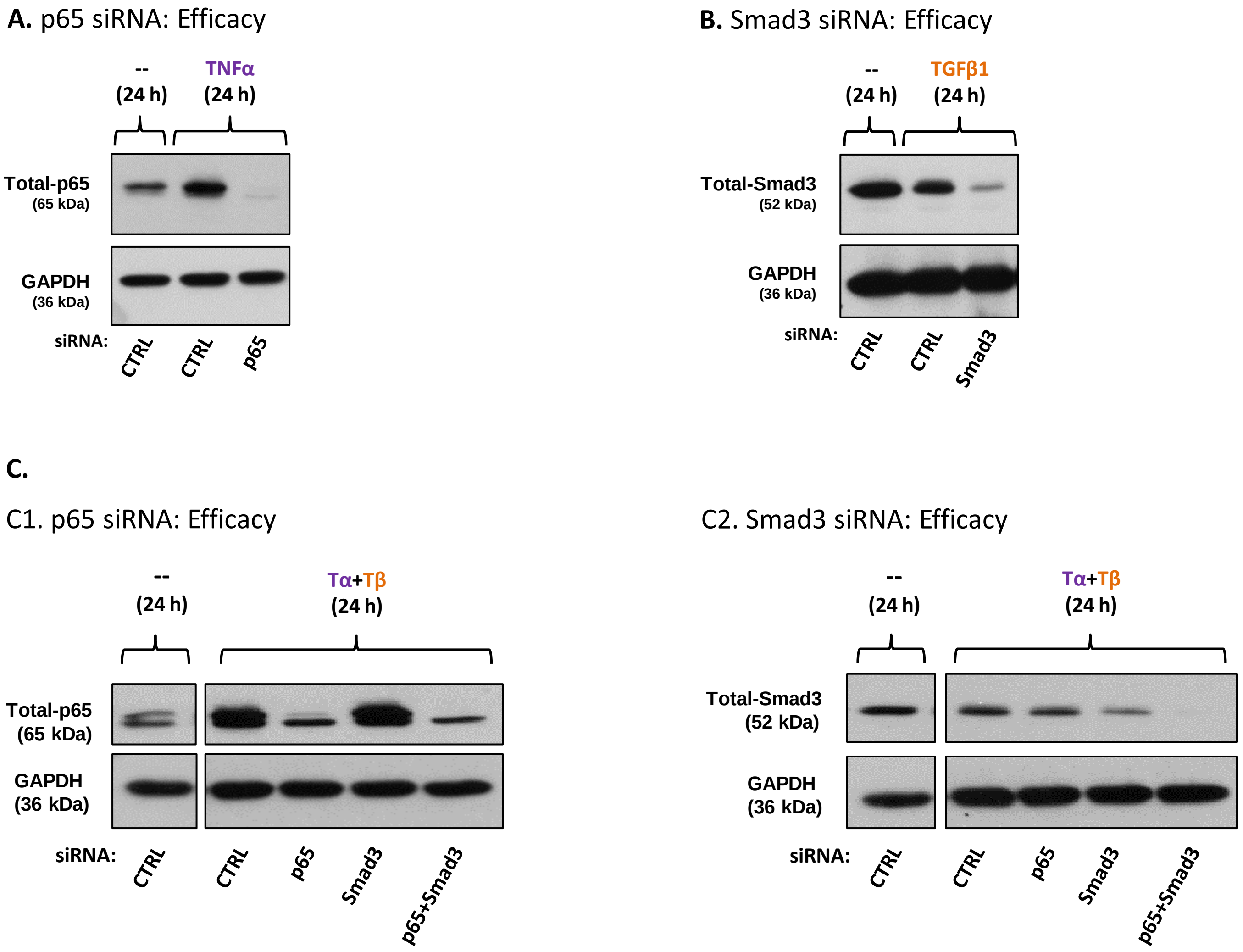

Supplementary Figure 4

Efficacy of p65 or Smad3 down-regulation following siRNA transfection

The figure complements the data of Figure 6 by demonstrating the efficacy of siRNA-mediated down-regulation of p65 and Smad3 expression, as appropriate. **(A)** Efficacy of p65 down-regulation by p65 siRNA, in TNFα-stimulated MSCs used in Figure 6A. **(B)** Efficacy of Smad3 down-regulation by Smad3 siRNA, in TGFβ1-stimulated MSCs used in Figure 6B. **(C)** Efficacies of p65 and Smad3 down-regulations by siRNA to p65 and Smad3, in TNFα+TGFβ1-stimulated MSCs used in Figure 6C.

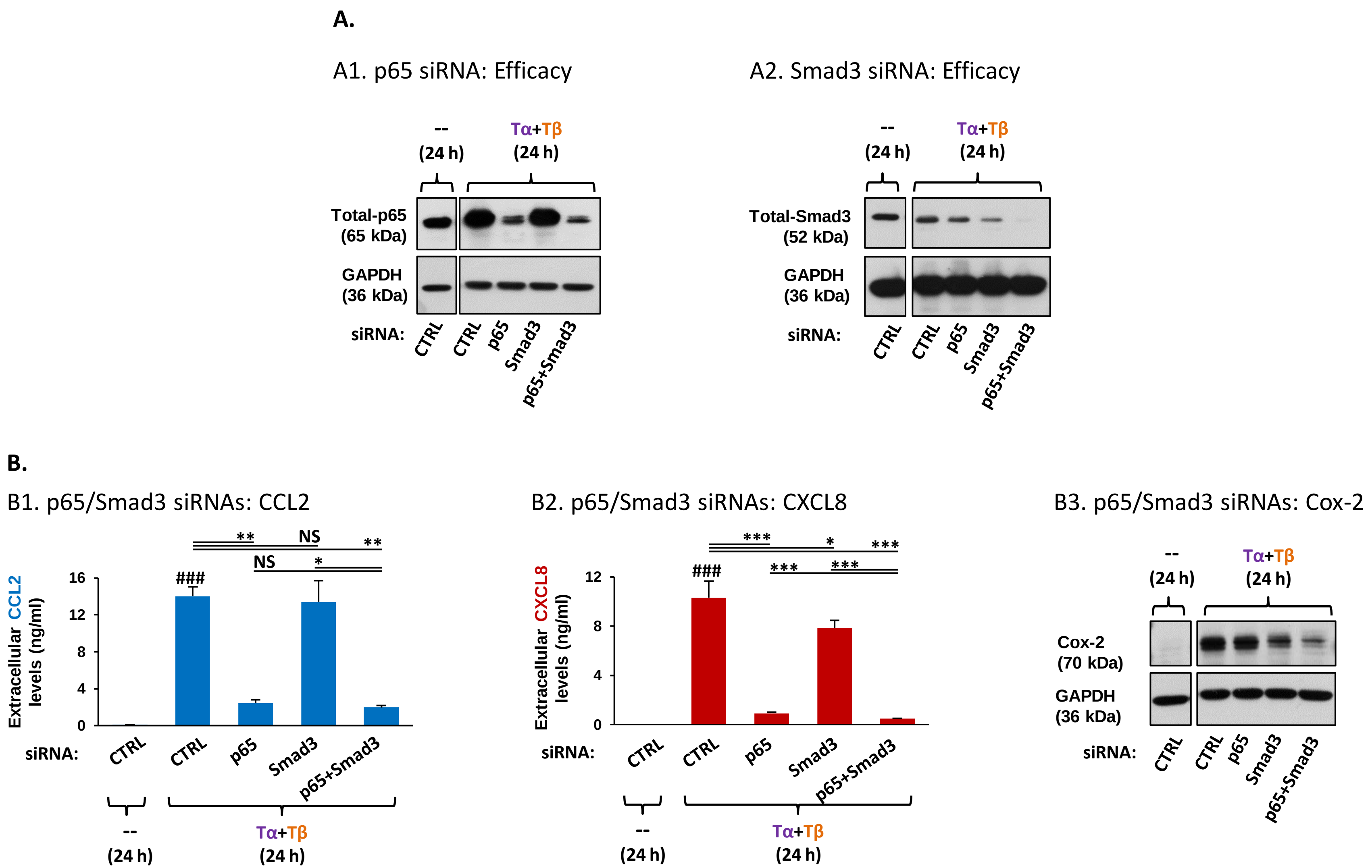

Supplementary Figure 5

Roles of p65 and Smad3 in regulating TNFα+TGFβ1-cooperatively-induced CCL2/CXCL8 and Cox-2 expression in MSCs – Another donor

The figure complements the findings presented in Figures 6C and Supplementary Figure 4C, by showing similar findings in another donor (out of total n=3 independent experiments). The different experimental parts of the figure were performed in parallel, using MSCs of the same donor. *p≤0.05, **p≤0.01, ***p≤0.001. ###p≤0.001 compared to vehicle-treated, control siRNA-transfected cells. NS, Not Significant.

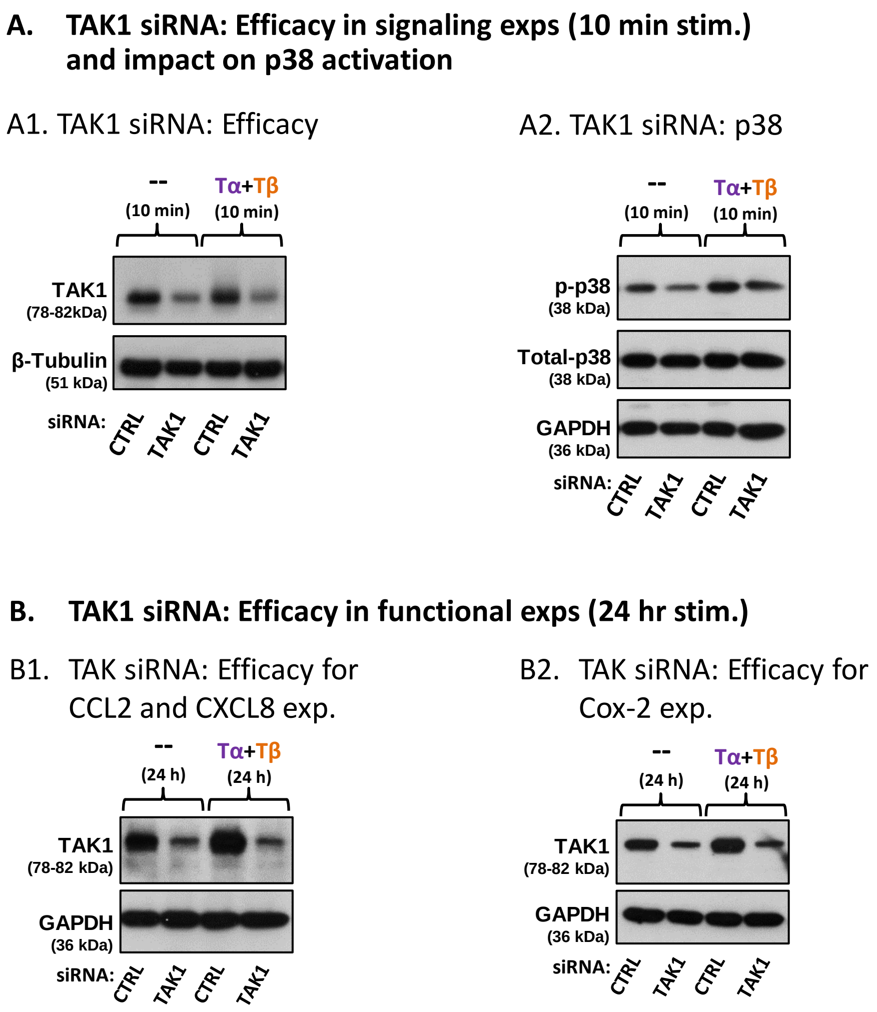

Supplementary Figure 6

The impact of TAK1 down-regulation on p38 activation and efficacy of TAK1 siRNA in the different relevant experiments

The figure complements the data of Figure 7. **(A)** TAK1 siRNA in signaling experiments. (A1) Efficacy of TAK1 down-regulation in TNFα+TGFβ1-stimulated MSCs used in Figure 7B (IκBα expression and p65 and Smad3 activation). Following the siRNA transfection procedure, MSCs were stimulated by the cytokines for 10 minutes. (A2) Following TAK1 down-regulation by siRNA, as demonstrated in Supplementary Figure 6A1, p38 activation was determined in the same MSCs. The findings are representatives of n=3 independent experiments, performed with MSCs of 2 different donors, which have shown similar results. **(B)** TAK1 siRNA in functional assays in which following siRNA transfection, the MSCs were stimulated for 24 hours with TNFα+TGFβ1 (no rapid signaling events are expected at this later time point). The findings were obtained with the MSCs used in Figure 7C. (B1) TAK1 down-regulation in analyses of CCL2 and CXCL8 expression (shown in Fig. 7C1, 6C2). (B2) TAK1 down-regulation in analyses of Cox-2 expression (shown in Fig. 7C3).

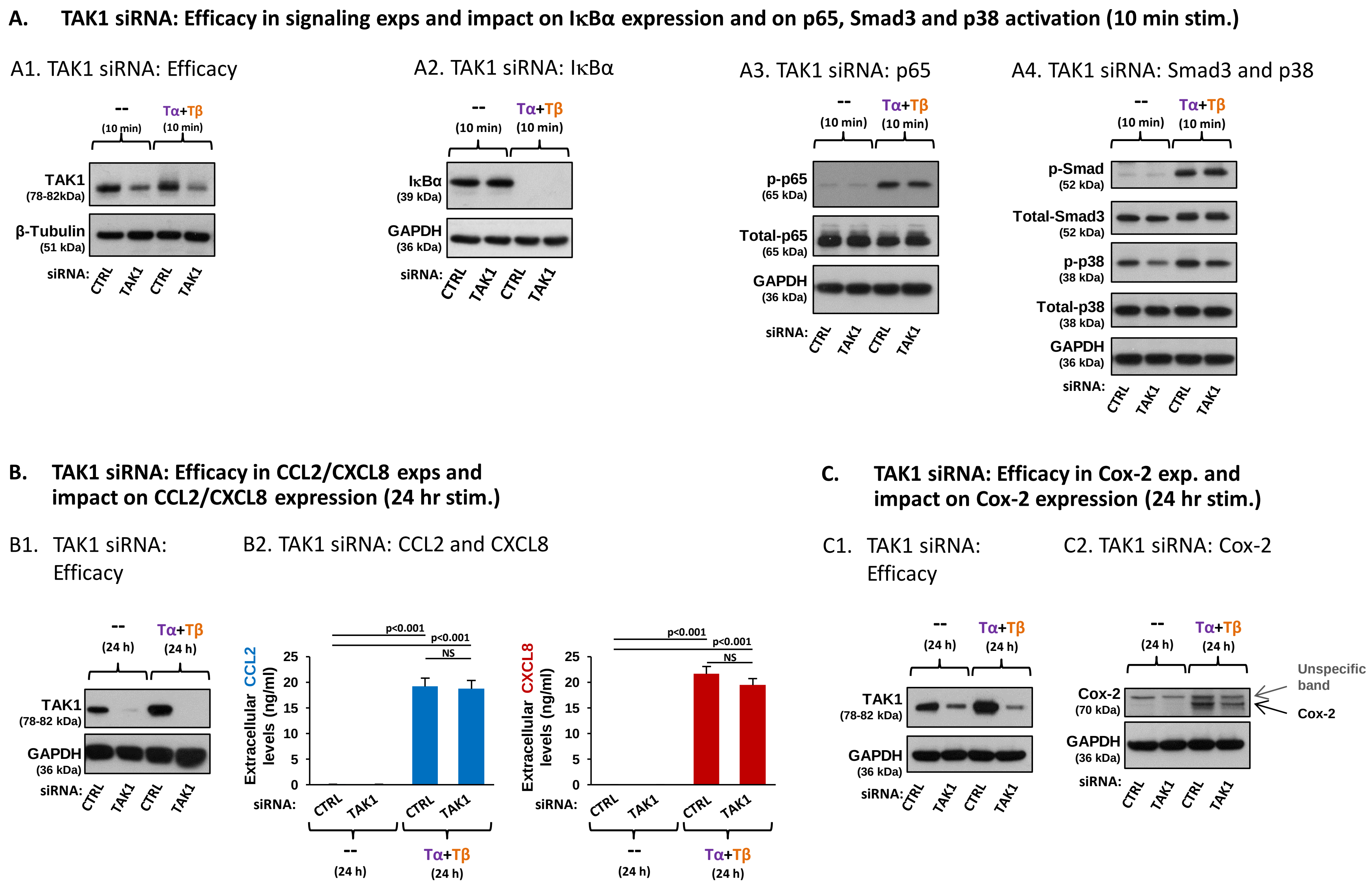

Supplementary Figure 7

Roles of TAK1 in regulating TNFα+TGFβ1-cooperatively-induced CCL2/CXCL8 and Cox-2 expression in MSCs – Another donor

The figure complements the findings presented in Figure 7 and in Supplementary Figure 6, by showing similar findings in another donor (out of total n=3 independent experiments). The different experimental parts of the figure were performed in parallel. More details are provided in the legends of Figure 7 and of Supplementary Figure 6 (note details about stimulation time).

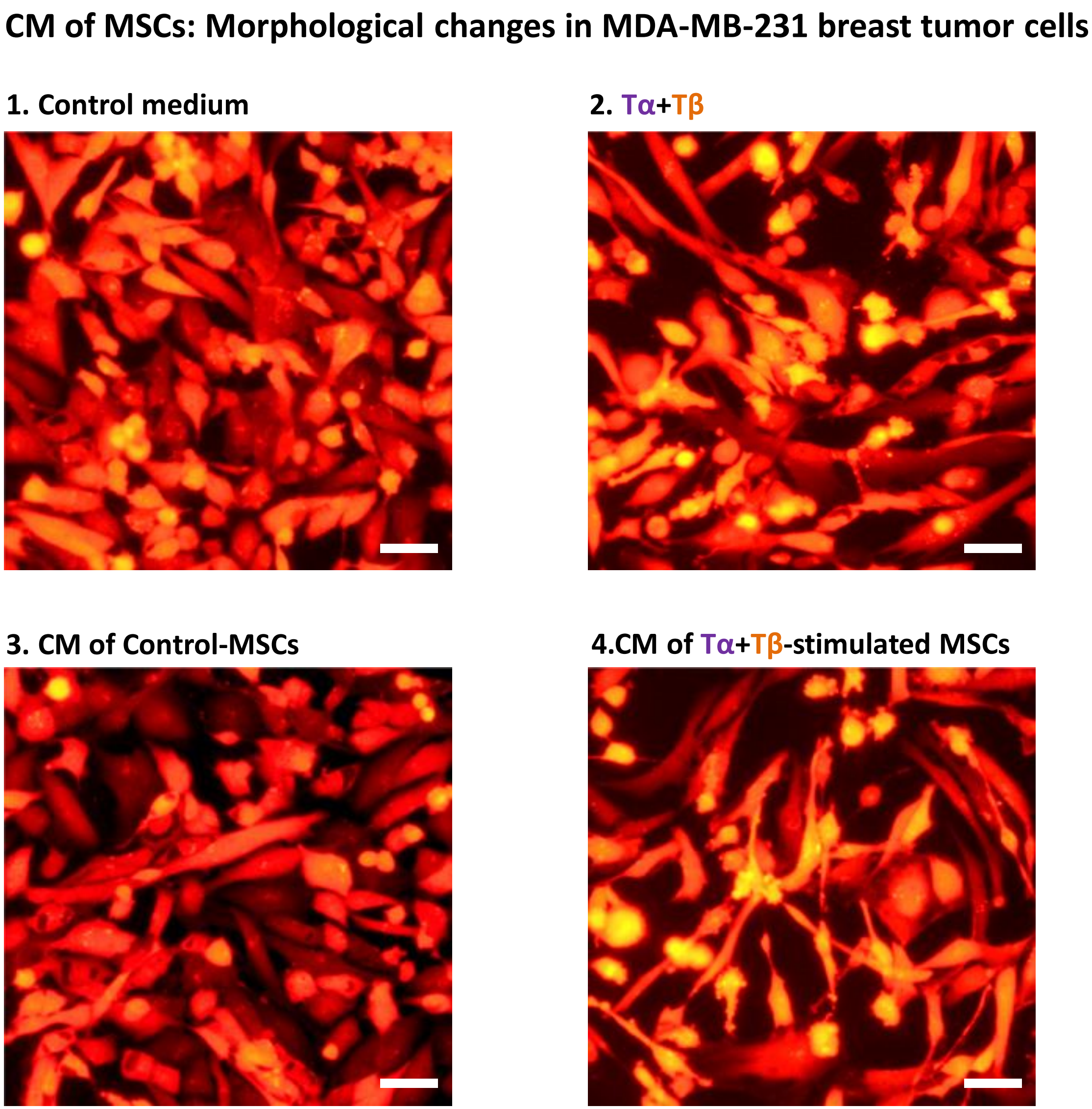

<u>Supplementary Figure 8</u>

Factors released by TNFα+TGFβ1-stimulated MSCs induce cellular elongation in MDA-MB-231 breast cancer cells – Another experiment

The figure complements the findings of Figure 8A by showing higher magnification of cells (Scale bar = 50μm), in another independent experiment (out of total n>3 independent experiments).

			3h		7h		14h		24h	
**GO.bp ID**	**GO.bp Term**	**Size**	**TNFα**	**TGFβ1**	**TNFα**	**TGFβ1**	**TNFα**	**TGFβ1**	**TNFα**	**TGFβ1**
GO:0006351	transcription- DNA-templated	1838	2.34E-16	1.97E-11	1.35E-13	1.44E-14	3.10E-05	3.73E-07		
GO:0044281	small molecule metabolic process	1483	2.72E-03	8.49E-03	6.52E-10	1.90E-09	4.73E-16	1.39E-12	1.93E-20	2.23E-12
GO:0006355	regulation of transcription- DNA-templated	1312	1.22E-07	2.55E-06	4.22E-05	5.40E-07		1.56E-03		
GO:0007165	signal transduction	988			5.03E-03		5.25E-05	1.71E-04	1.94E-03	2.29E-04
GO:0045944	positive regulation of transcription from RNA polymerase II promoter	969	7.01E-10	7.22E-11	2.44E-11	2.26E-12	7.07E-05	7.85E-10	1.31E-04	5.54E-05
GO:0010467	gene expression	881	1.81E-07	2.20E-08	9.70E-15	2.73E-21	6.36E-12	4.80E-16	1.87E-07	1.55E-07
GO:0045087	innate immune response	838			1.99E-07	8.71E-03		2.46E-03		
GO:0007186	G-protein coupled receptor signaling pathway	837	3.57E-07		7.59E-06	1.62E-10	1.06E-05	4.18E-04		3.30E-04
GO:0000122	negative regulation of transcription from RNA polymerase II promoter	725	5.66E-09	1.79E-06	3.33E-08	1.91E-11	6.80E-03	7.79E-05		
GO:0007264	small GTPase mediated signal transduction	653	8.64E-05	8.73E-06	8.27E-04	8.71E-11		4.04E-04		
GO:0044267	cellular protein metabolic process	640	2.99E-03		1.35E-10	9.17E-11	4.02E-10	6.56E-15	8.95E-05	3.76E-12
GO:0006915	apoptotic process	610	6.61E-05	1.25E-04	4.69E-09	3.41E-08	4.69E-09	2.78E-09	1.61E-08	2.24E-04
GO:0055114	oxidation-reduction process	582				1.31E-03	8.20E-04	6.29E-06	9.74E-06	2.28E-07
GO:0016032	viral process	566	4.06E-06		1.82E-09	2.56E-09	6.55E-08	2.92E-14	3.41E-07	1.98E-05
GO:0006366	transcription from RNA polymerase II promoter	554	9.31E-09	4.60E-04	2.34E-05	7.37E-08	1.50E-03	1.33E-05		3.42E-03
GO:0007411	axon guidance	540			7.39E-03	1.45E-06				7.42E-03
GO:0045893	positive regulation of transcription- DNA-templated	511	1.42E-06	2.67E-08	2.12E-06	1.74E-08	9.19E-05	2.76E-04		
GO:0043066	negative regulation of apoptotic process	486	9.08E-05		1.51E-07	4.26E-09	2.67E-09	1.72E-04	8.90E-06	
GO:0043547	positive regulation of GTPase activity	485	2.02E-03	2.32E-03						
GO:0045892	negative regulation of transcription- DNA-templated	482	6.85E-04		1.14E-06	5.91E-03		2.53E-05		
GO:0000278	mitotic cell cycle	434	1.85E-06	1.19E-04	1.12E-09	8.76E-12	1.91E-08		1.09E-12	
GO:0048011	neurotrophin TRK receptor signaling pathway	397			1.96E-08	5.00E-07				
GO:0050911	detection of chemical stimulus involved in sensory perception of smell	380	1.62E-08		4.68E-07	1.39E-12	5.07E-08	2.01E-09	1.12E-04	3.34E-04
GO:0016567	protein ubiquitination	363	2.34E-03	1.51E-03	6.83E-03	2.84E-07		2.17E-05		
GO:0008283	cell proliferation	355	2.34E-03		6.50E-03	5.05E-06	1.58E-04	2.34E-03	2.30E-04	
GO:0015031	protein transport	346			1.35E-06	1.07E-06	8.20E-04	1.39E-04	1.92E-03	6.53E-06
GO:0051301	cell division	331	2.55E-05		2.77E-04	2.16E-07		1.27E-06	2.72E-07	
GO:0007173	epidermal growth factor receptor signaling pathway	324			1.74E-04	1.65E-03				
GO:0043687	post-translational protein modification	316			4.33E-05	9.58E-03				7.32E-05
GO:0030198	extracellular matrix organization	313					8.47E-05	3.92E-04	4.04E-07	
GO:0038095	Fc-epsilon receptor signaling pathway	301			2.79E-04	2.17E-03				
GO:0006996	organelle organization	298	4.09E-03			4.91E-05	7.77E-05	1.37E-07	7.32E-03	4.80E-05
GO:0006281	DNA repair	292	2.81E-06	1.96E-04	3.02E-03	2.21E-06		7.40E-04	8.28E-05	
GO:0008543	fibroblast growth factor receptor signaling pathway	291			1.54E-04	1.35E-03				
GO:0048010	vascular endothelial growth factor receptor signaling pathway	284			3.38E-03	3.41E-04				
GO:0043065	positive regulation of apoptotic process	281	6.79E-05	6.23E-03	6.44E-08	4.30E-05	1.14E-05	1.87E-06	5.72E-04	
GO:0006367	transcription initiation from RNA polymerase II promoter	260				2.08E-03		2.27E-03	4.00E-03	
GO:0008380	RNA splicing	251	7.12E-03		1.08E-04	7.91E-11		8.79E-04		
GO:0007265	Ras protein signal transduction	244			8.21E-03	3.88E-03				
GO:0007067	mitotic nuclear division	241	1.62E-05		7.67E-04	1.39E-03		1.33E-05	5.65E-07	
GO:0006886	intracellular protein transport	235			8.58E-04	2.21E-06		1.52E-09		2.99E-03
GO:0000186	activation of MAPKK activity	232			3.09E-03	1.66E-03				
GO:0001525	angiogenesis	227			9.53E-03	4.11E-03		5.66E-04		
GO:0001701	in utero embryonic development	216	1.09E-04	1.74E-03	1.03E-03	3.07E-06				
GO:0007608	sensory perception of smell	213	3.80E-07		1.53E-03	4.91E-05	1.92E-04	6.45E-03		
GO:0000398	mRNA splicing- via spliceosome	209			1.39E-03	1.33E-09		1.51E-07		1.33E-09
GO:0061024	membrane organization	159			6.88E-03	2.90E-05		4.91E-05		1.08E-04
GO:0000082	G1/S transition of mitotic cell cycle	155			1.74E-04	3.01E-04	1.10E-04			
GO:0044255	cellular lipid metabolic process	153				1.85E-03	4.38E-04	1.66E-04	1.61E-04	2.15E-04
GO:0000086	G2/M transition of mitotic cell cycle	141			3.02E-03	1.65E-03			1.60E-03	
GO:0006260	DNA replication	134		8.50E-03	9.50E-04	7.71E-03	1.51E-03			
GO:0030968	endoplasmic reticulum unfolded protein response	101	1.46E-03		5.21E-06	5.48E-05	1.40E-03	5.19E-05		7.72E-04
GO:0002474	antigen processing and presentation of peptide antigen via MHC class I	99			2.15E-03	8.54E-03				
GO:0006364	rRNA processing	94				3.85E-06	9.40E-04	3.92E-04		4.08E-03
GO:0034138	toll-like receptor 3 signaling pathway	81	9.90E-03		5.75E-06	9.10E-03	2.96E-03			
GO:0031398	positive regulation of protein ubiquitination	63	6.05E-03			9.47E-03				
GO:0036498	IRE1-mediated unfolded protein response	55			5.96E-03					3.42E-03

Supplementary Table 1

Kinetics-based shifts in transcriptional programs induced by both TNFα and TGFβ1 in MSCs

Gene ontology (GO) enrichment analysis of pathways that were modified by both TNFα and TGFβ1 at one time point or more ($p \leq 0.01$, after BY correction for multiple-testing, compared to vehicle-treated cells). The table indicates the p values of change of each GO term, at each specific time point following stimulation by TNFα or TGFβ1. White squares – The response did not pass the required cut-off. Size = Number of genes that are included in the transcriptional program.

Supplementary Table 2:
Primers used in qPCR analyses

Supp. Table 2A

Gene	Primer direction (fw=forward, rv=reverse)	Sequence (5' -> 3')
PTGS2	PTGS2_fw	actctggctagacagcgtaa
	PTGS2_rv	agatcatctctgcctgagtatc
CX3CL1	CX3CL1_fw	ccaccttctgccatctgac
	CX3CL1_rv	atgttgcatttcgtcacacc
EPSTI1	EPSTI1_fw	aggcaaaagtcaaccaggtg
	EPSTI1_rv	tgaaggccagataggagtcaa
ANGPTL4	ANGPTL4_fw	gtggaccctgaggtccttc
	ANGPTL4_rv	ccaccttgtggaagagttgc
PTHLH	PTHLH_fw	ctcggtggagggtctcag
	PTHLH_rv	tggatggacttccccttgt
PLAU	PLAU_fw	ttgctcaccacaacgacatt
	PLAU_rv	ggcaggcagatggtctgtat
RPS9	RPS9_fw	aacttatgtgaccccgcgga
	RPS9_rv	cagcttcagctcttggtcga

Supp. Table 2B

Gene	Primer direction (fw=forward, rv=reverse)	Sequence (5' -> 3')	UPL Probe
CCL2	CCL2_fw	agtctctgccgcccttct	#40
	CCL2_rv	gtgactggggcattgattg	
CXCL8	CXCL8_fw	agacagcagagcacacaagc	#72
	CXCL8_rv	atggttccttccggtggt	
NGF	NGF_fw	tccggacccaataacagttt	#32
	NGF_rv	ggacattacgctatgcacctc	
IL6	IL6_fw	gatgagtacaaaagtcctgatcca	#40
	IL6_rv	ctgcagccactggttctgt	
LIF	LIF_fw	tgccaatgccctctttattc	#26
	LIF_rv	gtccaggttgttggggaac	
HBEGF	HBEGF_fw	tggggcttctcatgtttagg	#55
	HBEGF_rv	catgcccaacttcactttctc	
CSF2	CSF2_fw	tctcagaaatgtttgacctcca	#1
	CSF2_rv	gcccttgagcttggtgag	

MMP1	MMP1_fw	gctaacctttgatgctataactacga	#7
	MMP1_rv	tttgtgcgcatgtagaatctg	
MMP3	MMP3_fw	caaaacatatttctttgtagaggacaa	#36
	MMP3_rv	ttcagctatttgcttgggaaa	
VEGFC	VEGFC_fw	tgccagcaacactaccacag	#27
	VEGFC_rv	gtgattattccacatgtaattggtg	
FGF1	FGF1_fw	accaagtggattctgcttcc	#56
	FGF1_rv	cttgtggcgctttcaagac	
IL12A	IL12A_fw	cactcccaaaacctgctgag	#50
	IL12A_rv	caatctcttcagaagtgcaagg	
GAPDH	GAPDH_fw	agccacatcgctcagacac	#60
	GAPDH_rv	gcccaatacgaccaaatcc	
HPRT	HPRT_fw	tgaccttgatttattttgcatacc	#73
	HPRT_rv	cgagcaagacgttcagtcct	
